# A Self‐Monitoring Mobile App to Mitigate Risk Factors for Suicide and Self‐Harm in Junior (Resident) Doctors: A Review, Thematic Analysis and Concept Proposal

**DOI:** 10.1049/htl2.70009

**Published:** 2025-05-06

**Authors:** Kirsten Leslie, Chloe Sawyer, Katy Oak, Gareth Lewis, Bryan Clark, Anna Mankee‐Williams, Ellen Wilkinson, Hiu Lam, Richard Laugharne, Rohit Shankar

**Affiliations:** ^1^ Royal Cornwall Hospital Trust, Truro Cornwall UK; ^2^ Cornwall Partnership NHS Foundation Trust Cornwall UK; ^3^ Falmouth University, Falmouth Cornwall UK; ^4^ Health Education England Plymouth UK; ^5^ Cornwall Intellectual Disability Equitable Research (CIDER) University of Plymouth, Health and Wellbeing Innovation Centre, Truro Cornwall UK

**Keywords:** digital app, doctors mental health, mental stress, self‐harm, self‐injury, suicide ideation

## Abstract

Doctors, particularly those in training in the UK, are exposed to high levels of stress in their work, which can lead to burnout and mental health problems. According to the health and safety executive (HSE) Management UK standards, employers should recognise and minimise work‐related stress for staff. Our review looks to examine if known risk factors for suicide and self‐harm in doctors align with the themes of the HSE management standards on stress control i.e., demand, control, support, relationships, role, and change and if so, could this be used to build a self‐awareness digital application. Four research databases were searched using combinations of text words and thesaurus terms and predefined inclusion/exclusion criteria for relevant article retrieval. A thematic analysis was undertaken, aligning articles to their respective HSE standards. Twenty‐six articles met the full inclusion criteria. 96.2% (25/26 papers) mentioned or aligned at least one of the HSE management standards, with 44% discussing three or more. Work‐related risk factors for self‐harm and suicide in doctors link well to the HSE management standards. We conceptualise a self‐monitoring digital well‐being tool for doctors to monitor stress.

## Abstract 2

Our review looks to examine if known risk factors for suicide and self‐harm in doctors, align to the themes of the HSE management standards on stress control i.e., demand, control, support, relationships, role, and change and if so, could this be used to build a self‐awareness digital application. Work‐related risk factors for self‐harm and suicide in doctors link well to the HSE management standards. We conceptualise a self‐monitoring digital well‐being tool for doctors to monitor stress, workplace tensions, and by extension mitigate mental health concerns.

## 1 Introduction

Doctors’ suicide rate is two to five times higher than the general population [1]. Though concerns have always existed about doctor suicides in the UK, it has lately increased due to rising suicide rates among junior doctors as opposed to the recognised demographic of established doctors [2]. Evidence suggests that the higher risk of suicide in doctors is a multifaceted issue with underpinnings linked significantly to their workplace culture and support. This is an area that needs greater recognition, as at present it remains nebulous and there is a lack of an evidence‐based synthesis of the various cumulative factors influencing negative outcomes which undoubtedly has an impact on the overall picture.

The transition from medical student to junior doctor has long been considered a significant rite of passage and is frequently experienced as stressful [3, 4, 5, 6]. Between 1993 and 2012 a multipurpose survey of UK post graduate doctors focussing on doctors working when acutely ill was undertaken at postgraduate years one, five and ten [7]. They found that the symptoms of stress and exhaustion were highest in the first year [7]. In addition, studies and reviews have shown a substantial minority of doctors go on to develop mental health conditions throughout their career, e.g., depression (20–25%), addiction (10%), burnout (up to 80%) and higher suicidal thoughts or action to kill themselves [8, 9, 10]. The findings are of course, based on the study population, country of study, nature of service, type of speciality and time of study [8]. Research shows that certain personality characteristics are consistent with better well‐being [11]. Practices such as mindfulness, coaching, and mentoring and part‐time employment have also been found to be helpful in maintaining well‐being [12, 13, 14]. It has been suggested that there is a poor understanding of how best to emotionally support doctors in general and new doctors in particular to sustain their mental well‐being, reduce their suicide risk, and address any factors that might be associated with these phenomena [15]. However, a recent systematic review suggests that a combination of workplace, personal, and non‐workplace factors positively influence wellbeing and resilience levels [16]. As stress and well‐being are multifactorial and highly individualised, it is likely that although resources exist, individuals are either not aware of them or may lack a self‐awareness of the issues.

Completed suicide is a tragic occurrence, characterised by the result of various cumulative events which have impacted negatively on an individual's experience of the world. While any suicide is a tragedy, the occurrence of this in young, highly trained and motivated doctors is a significant concern. In the UK, 81 doctors completed suicide 2011–2015 [17]. Each year in America, 300–400 physicians end their lives, which is approximately the size of a graduating class [18]. This raises multiple questions including how best to help the vulnerable population self‐realise their risks. Historically, there is variance among doctors based on sex and profession thus making it difficult to have a single strategy for doctors [19, 20, 21].

The issue of how to provide support and treatment interventions for doctors experiencing mental health difficulties and their related impacts has been the topic of numerous academic works [22, 23, 24]. Often, workplace stress management interventions focus on increasing the individual doctors’ ‘efficacy’ or ‘resilience’ [24]. However, the charter for psychological staff well‐being and resilience and the well‐being collaborative for learning network has directed employers to put the necessary means to promote wellbeing in the workplace through reflective practice and compassionate cultural workplaces [25]. In the context of the HSE management standards this could arguably be perceived as abdicating organisational responsibility regarding some known workplace stressors, preferring to let mental well‐being rest with doctors themselves [24, 26, 27, 28, 29, 30]. A recent systematic review has shown that the HSE management standards show good reliability as a measure of possible causes of work‐related stress [31]. Although no papers on doctors were found by this review, they did include data from hospice employees and nursing and allied health professionals working in mental health settings and veterinary surgeons [31].

There has been limited focus in the medical arena on active organisational support, interventions, and systemic factors contributing to mental ill‐health, and more on individual doctors [24, 29], which demonstrates the necessity of exploring ‘organisational resilience’ [24, 28]. According to the HSE management standards (Supplementary Information 1), employers have a legal duty to minimise work‐related stress, recognise where stress is becoming a problem for staff, and take action to reduce stress by undertaking a risk assessment [30].

Long‐term stress reduces productivity, increases management pressures, and generally makes people ill [30]. Stress at work also provides a serious risk of litigation for all employers, as well as bad publicity, and consequent loss of reputation [30]. There is recognition that doctors’ roles are stressful. The HSE management standards define stress as ‘the adverse reaction people have to the excessive pressures or other types of demand placed upon them’ [30]. Given each individual is different and has a different set of professional and personal coping mechanisms, gaining an evidence‐based understanding of influencing factors at a personal and organizational level can be challenging [24].

## 2 Aim

To see if the currently known risk factors for suicide and self‐harm in doctors align with the themes of the HSE management standards on stress control.

## 3 Methods

A search strategy, which followed the PRISMA scoping review guidance Appendix 1, was constructed that utilised combinations of text words and thesaurus terms for the retrieval of articles relating to doctors and suicide in collaboration with two information specialists. The following limits were applied to the search: English language, date range 2007 to 2018, with the last search being conducted on 07/11/2018. This search was conducted on embase, medline, PsycINFO (Appendix 2). Supplementary searches were carried out on the cochrane library for cochrane systematic reviews.

Following the removal of duplicates, articles were screened by title and abstract, and then by full text for relevance to the pre‐identified criteria (Appendix 2). Articles were included if: they were original research/systematic reviews, and identified risk factors for suicide or self‐harm in physicians or doctors in training. Articles were excluded if: they pertained to other professional groups, were based on opinion, did not focus on risk factors for suicide, or self‐harm, suicidality was assessed by a proxy measure such as hopelessness or results on suicidality could not be clearly separated from those on other factors. Articles were assessed on various parameters, particularly the number and type of HSE standards each satisfied.

## 4 Results

Twenty‐six articles met the inclusion criteria, and their full texts were analysed. The relevant findings are presented in Table 1. These included a systematic r/w, two retrospective cohort studies, one case‐control study, 21 cross‐sectional studies and one case report. The studies, when grouped into three yearly periods were three from 2007–2009, four from 2010–2012, 10 from 2013–2015, and nine from 2016–2018.

**TABLE 1:** Currently known risk factors for self‐harm and suicide in physicians and how they align to the HSE Management Standards.

Study	Participants	Risk factors identified	HSE management standards	Study outcomes
Oskrochi et al. [43] Systematic review	71 articles on non‐physical effects of surgical career	Age Speciality Gender Depression Positive screen Burnout Divorce Recent Medical error Number of nights on call Malpractice Lawsuit Alcohol abuse or dependence	Demand relationships control support	Surgeons aged 45–54, 55–64, and ≥64 were 1.5‐3x more likely to have suicidal ideation than the general population [42]. The highest rates of suicidal ideation were associated with vascular, cardiothoracic, and trauma surgeons. Female physicians have over double the risk of suicidal ideation compared to the general population. Risk factors for suicidal ideation also included: depression positive screen, burnout, divorce, recent medical error [42], number of nights on call, recent experience of a malpractice lawsuit, and alcohol abuse or dependence. Protective factors: being married or in a relationship, having young children, working in an academic centre [42].
Agerbo et al. [47] Case‐control	36 Danish physicians 25–60 years old who died by completed suicide between 1991–1997	Occupation access to means mental illness	Role relationships	Doctors had the highest relative risk of suicide (RR 2.73 CI 1.77–4.22 p < 0.05) in comparison to primary school teachers (reference population). Relative risk increased when adjusted for confounding factors. Doctors had a significantly increased risk of positioning by medical compounds (RR 11.12, CI 5.98–20.66, *p* < 0.0001). Being admitted to hospital with a psychiatric disorder put doctors at 3.62x increase risk of completed suicide (CI 1.43–9.15, *p* < 0.05).
Skegg et al. [61] Retrospective cohort	27 physicians in New Zealand that completed suicide between 1973–2004	Access to means	0	Physicians had a significant positive association with poisoning as a method for completed suicide. Doctors were not at increased risk of suicide in this study compared to the general population of New Zealand. Male physicians appeared to have a significant negative association with suicide.
Braquehais et al. [62] Retrospective cohort	21 physicians (and 18 nurses) admitted to an inpatient unit for health professionals in Barcelona.	Age Gender Previous Suicide attempt Depression Personality traits Psychosocial stressors	Support	Physicians with a recent suicide attempt (RSA) were younger than those who hadn't attempted suicide. Female physicians were more likely to have attempted suicide than males (OR 3.252, CI 1.562–6.770, *p* < 0.05). Patients with RSA were more likely to have a history of suicide attempts, unipolar depression, and personality disorders (cluster B or C) than those without a RSA. The doctors were not separated from nurses in the statistical analysis. Physicians made more lethal attempts than nurses and were significantly more likely to have > 1 psychosocial and environmental problems prior to their suicide attempt.
Dyrbye et al. [45] Cross‐sectional	13391 American medical students, residents and early career physicians	Stage of career	Support Demand	Medical students were significantly more likely to have suicidal ideation than residents or early career physicians. (*p* = 0.0058) Marriage had a significant negative association with suicidal ideation (*p* < 0.001)
Bourne et al. [39] Cross‐sectional	7296 BMA members	Complaints GMC Investigations	Support Relationships Control	Physicians with recent or current complaints were twice as likely to have thoughts of self‐harm. People undergoing GMC referral reported the most thoughts of self‐harm.
Shanafelt et al. [42] Cross‐sectional	7905 American surgeons	Age Divorce Working > 40 h per week Number of overnight on‐calls Perceived medical error Burnout Depression	Support Demand Relationships Role	Surgeons aged 45–54, 55–64, and ≥65 had a significant positive association with suicidal ideation. Surgeons aged 55–64 were 3x more likely than the general population to have suicidal ideation (*p* < 0.001). Divorce had a significant positive association to suicidal ideation in the past year (OR 1.634 1.337–1.995, *p* < 0.001). Suicidal ideation in surgeons had a significant positive association with working > 40 h per week, higher numbers of overnight on‐calls, perceived major medical error in the preceding three months, burnout, and depression. Having children, being married, working in an academic centre, and practicing for > 30 years were significantly negatively associated with suicidal ideation.
Dyrbye et al. [37] Cross‐sectional	6994 American physicians	Well‐being	Demand Support	Recent suicidal ideation increased the likelihood of physicians scoring on each item on the physician Well‐being index (*p* < 0.001). Increasing scores on the PWBI were not significantly associated with the odds of suicidal ideation.
Tawfik et al. [41] Cross‐sectional	6586 US physicians in active practice	Medical errors	Support	Significant positive association with self‐perceived medical errors in the past three months.
Wada et al. [40] Cross‐sectional	3864 Japanese physicians	Complaints Demands	Demand Support Relationships	Significant positive association with four or more complaints or unreasonable demands in past six months.
Mullola et al. [63] Cross‐sectional	2815 Finnish specialists and specialising physicians	Personality traits	Relationships Demand Control	High neuroticism, high openness to experience, low agreeableness and low extraversion all had significant positive associations with suicidal ideation. No effect of gender, but some effect of speciality on suicidal ideation demonstrated.
Dyrbye et al. [38] Cross‐sectional	1701 American residents and fellows	Well‐being	Support	Recent suicidal ideation increased the likelihood of physicians scoring on each item on the physician well‐being Index (*p* < 0.001). Increasing scores on the PWBI were not significantly associated with increasing odds of suicidal ideation.
Rosta et al. [50] Cross‐sectional	Approx. 1600 Norwegian physicians and 1917 German doctors.	Psychosocial work stress Physical Health Gender Speciality	Support Change Demand Role Relationships	Significant reduction in suicidal feelings between 2000–2010 (paired data) for Norwegian physicians. Poor/average self‐rated health (OR 2.36, CI 1.25–4.45, *p* = 0.008), high levels of psychosocial stress at work (OR 1.92, CI 1.06–3.46, *p* = 0.031), subjective wellbeing (OR 0.68, CI 0.52–0.90, *p* = 0.007) all had significant positive association with suicidal feelings for Norwegian physicians. Working in psychiatry and female gender both had significant positive associations with suicidal feelings when comparing Norwegian and German physicians.
De Oliveira et al. [64] Cross‐sectional	1384 American anaesthesiologists	Depression	Demand Control Support	Screening positive for depression had a significant positive association with suicidal ideation in the last two weeks.
Eneroth et al. [35] Cross‐sectional	1047 Swedish physicians (residents and specialists) from a university hospital	Sickness presenteeism Harassment Disengagement at work	Demand Control Support Relationships Role Change	Sickness presenteeism and disengagement at work had significant positive associations for both specialists and residents. Harassment at work also had a significant positive association for specialists. Positive leadership had a significant negative association for residents. Regular meetings to discuss demanding experiences at work had a significant negative association for specialists.
Fridner et al. [34] Cross‐sectional	697 male physicians from Sweden (456) and Italy (241)	Degrading experiences/harassment Role conflict	Demand Support Role Control Relationships	Swedish physicians—role conflict and recent degrading experiences had significant positive associations. Support at work had a significant negative association. Italian physicians—only degrading experiences/harassment had a significant positive association. Control over working hours and amount of work allocated, and having confidential discussions about work experiences had significant negative associations with suicidal ideation.
Loas et al. [44] Cross‐sectional	557 currently licenced physicians or residents in Brussels	Gender Relationship Status Perceived burdensomeness Thwarted belongingness Anhedonia Lack of satisfaction Lack of interest Work inhibition Depression	Demand Relationships	The prevalence of women was significantly higher in physicians with recent suicidal ideation (*p* = 0.009) and lifetime suicidal ideation (*p* = 0.022) than those without. Physicians with lifetime suicidal ideation were significantly more likely to be single (*p* = 0.001). Perceived burdensomeness, thwarted belongingness, anhedonia, lack of satisfaction, lack of interest, work inhibition, and cognitive/affective symptoms of depression had a significant positive association with both lifetime and recent suicidal ideation. Thwarted belongingness, anhedonia, lack of satisfaction, work inhibition, and cognitive/affective symptoms of depression have a significant positive association with suicide attempts.
Lebares et al. [36] Cross‐sectional	556 general surgery residents	Burnout High Emotional Exhaustion High Depersonalisation High Stress	Role Change Support	Identified risk factors all had a significant positive association over the preceding two weeks. Dispositional mindfulness and high trait resilience had a significant negative association.
Bauer et al. [65] Cross‐sectional	419 Licenced physicians in Pennsylvania	Painful and provocative events Life events	Support	Scores on the life events scale—medical doctors (LES‐MD) and painful and provocative experiences scale (PPES) were significantly associated with scores on acquired capability for suicide scale. Acquired capability for suicide scale scores, LES‐MD scores, and PPES scores were not significantly associated with prior suicide attempts. No effect of age on ACSS scores was demonstrated in this study.
Fink‐Miller et al. [48] Cross‐sectional	376 licenced physicians in Pennsylvania	Provocative Work Experiences	Role	Frequency of provocative work experiences predicted acquired capability for suicide even when controlling for gender and provocative life experiences outside of work. Variance in acquired capability for suicide was not found between specialities Life events with positive association to acquired capability for suicide (*p* < 0.01) Resuscitation, performing surgery, witnessing serious physical trauma in a patient, witnessing an unexpected patient death, giving someone a life‐limiting diagnosis, discussing end‐of‐life issues with a patient or family, witnessing an expected patient death, and talking to a patient's family member about death or poor clinical outcomes.
Rath et al. [66] Cross‐Sectional	369 current senior, full or candidate members of American Society of Gynaecological Oncologists	Burnout	Role Demand Control	Physicians with a history of suicidal ideation had a significant positive association with burnout (OR 4.92, CI 2.59‐9.34, *p* < 0.001)
Lindfors et al. [33] Cross‐sectional	328 Finnish anaesthesiologists	Poor Health Lack of social support Depression Conflict with co‐workers Lack of organisational justice Taking antidepressants ≥6 hangovers in a year High on‐call stress Family problems Smoking	Demand Support Relationships Control	All risk factors listed had significant positive associations with lifetime suicidal ideation. Low social support, use of antidepressants, family problems, depression, and conflicts with co‐workers had the most significant associations. The risk of suicidal ideation doubled with each additional risk factor.
Pereira‐Llma et al. [32] Cross‐sectional	228 resident physicians from 30 residency programs	Protective factors only	Support Relationships	Feelings of belonging to a team and safety culture had a significant negative association with suicidal ideation. Conference abstract only.
Talih et al. [49] Cross‐sectional	118 residents from Beirut	Burnout Severe Depression Occupation	Change Relationships	Burnout and depression severity had a significant positive association with suicidal ideation. Residents with major depression were significantly more likely to have suicidal ideation than residents with mild depression. Burnout and stressful life events over the past 12 months were significant predictors of suicidal ideation. The rate of suicidal ideation 3x higher than general population of Lebanon.
Oumaya et al. [67] Cross‐sectional	113 Tunisian primary care physicians	Burnout	Relationships	Burnout was significantly positively associated with suicidality. Conference abstract only.
Colucci et al. [68] Case Report	1 Italian anaesthesiologist	Substance misuse	Relationships	A self‐administered cocktail of drugs to complete suicide, including propofol. Lab reports show evidence of recreational propofol misuse. A recent divorce from wife.

Twenty‐five of the 26 papers (96.2%) analysed in this review mentioned at least one of the HSE management standards, with 44% (12/26 papers) discussing three or more. Support was the most commonly assessed theme, with 17 papers discussing this. Other common themes were relationships (15 papers) and demand (13 papers), with control (8 papers*)*, role (8 papers), and change (4 papers) taking less of the focus.

### 4.1 Support

Support was the most acknowledged HSE Management Standard, with 18 papers making reference to it. Unsurprisingly, feelings of belonging to a team and a safety culture both had a significant negative association with suicidal ideation [32]. Low social support and a lack of organisational justice were shown to increase Finnish anaesthesiologist's risk of suicidal ideation by 10.7 and 1.8 times respectively [33]. The HOUPE study showed that being able to rely on others for support at work had a significant impact on suicidality for Swedish physicians, whereas confidential discussions about work experiences conveyed more protection for Italian physicians [34]. Another study found that positive leadership was shown to be protective against suicidal ideation for residents, while regular meetings to discuss demanding experiences were protective for specialists [35]. This infers that the type of support required varies not only by culture [34] but also by level of training [35]. Furthermore, several papers trailed methods to support physicians including dispositional mindfulness [36] and the Physician well‐being Index [37, 38]. Support was also the most likely standard to be referenced in discussions, being called upon to mediate the effects of complaints [39, 40] and medical errors [41, 42, 43], as well as other sources of inherent occupational stress.

### 4.2 Relationships

Relationships were shown to play a significant role in physician well‐being. Physicians with lifetime suicidal ideation were significantly more likely to be single, with divorce being demonstrated as a significant risk factor in American surgeons [42, 43, 44]. Moreover, conflict with co‐workers and family problems increased Finnish anaesthesiologist's risk of suicidal ideation by 3.9 and 5.8 times, respectively [33]. Experiences such as harassment at work were shown to have a significant positive association with suicidal ideation [34, 35]. Relationships were also shown to have a significant negative association with suicidal ideation, with marriage and children both being protective factors [42, 43, 45].

### 4.3 Demand

The occupational demands upon physicians were often cited in introductory parts of papers to add further weight to research topics. Several articles reviewed the effects of demand on suicidal ideation, with high on‐call stress, working more than 40 h per week, and the number of nights on call showing a significant positive association for physicians [33, 42, 43]. Having four or more complaints or unreasonable demands in the past six months increased odds of male and female Japanese physicians experiencing suicidal ideation by 2.25 and 4.56 times, respectively [40]. This has become such a problem in Japan that they have named it ‘karojisatsu’, suicide due to overwork [40, 46]. Furthermore, sickness presenteeism has also been linked to suicidal ideation which may be influenced by rota demands as well as fear of stigmatisation and a possible lack of self‐awareness [35].

### 4.4 Role

Like demand, role was often cited as a possible reason for decreased physician well‐being. Unsurprisingly, suicidal ideation appears to be its own occupational hazard for physicians. In a 2007 case‐control study, doctors were found to be almost three times as likely to complete suicide as primary school teachers [47]. This association increased on controlling for confounding factors of psychiatric admission, employment status, marital status, and gross income [47]. In one study, role conflict was shown to increase the suicidal ideation of Swedish physicians by 1.6 times [34]. Moreover, acquired capability for suicide was shown to have a significant positive association with the frequency of provocative work experiences in physicians, even when controlling for gender and provocative life experiences outside of work [48]. Some of the most significant provocative experiences for physicians were giving someone a life‐limiting diagnosis, performing surgery, and witnessing both expected and unexpected patient deaths [48].

### 4.5 Control

Despite several papers acknowledging it, the only article to assess control was the HOUPE study. It showed that having control over working hours and the amount of work allocated showed a significant negative association with suicidal ideation for Italian physicians [34].

### 4.6 Change

Change was the standard to be referenced least often. One study of residents in Beirut showed that burnout and stressful life events over the past 12 months were significant predictors of suicidal ideation [49]. Furthermore, paired data from Norwegian physicians in 2000 and 2010 showed a significant decrease in suicidal ideation [50]. One of the possible explanations for this change by the authors was the implementation of several health care reforms, which are thought to have led to improvements in working conditions [50].

## 5 Discussion

From the results, it can be seen that numerous risk factors, particularly stress have been identified for suicidal ideation and self‐harm in physicians. Twenty‐five of the 26 papers identified acknowledged at least one of the HSE management standards, and all six HSE standards were recognised to different degrees in the papers.

### 5.1 Limitations

This paper is limited by several factors. Firstly, although systematic review methodology was followed, the search only covers a specific time period (2007–2018) and databases. We have then looked to see any emerging literature in this area. In future, it would be prudent to also review any grey literature associated with this topic, as well as broadening our search criteria to include other indicators of poor well‐being such as burnout, addiction, depression, and anxiety. Furthermore, the majority of the papers analysed were cross‐sectional with poor response rates, which leaves them open to responder and recall bias. It also limits the interpretation of results as causality and temporality cannot be assumed. The HSE management standards were chosen due to their use of ease in providing a framework to measure workplace stress. However, it needs to be outlined that the HSE standards are organizational factors and not validated for individual use. Additionally, these factors are over a decade old. Many of the studies reviewed in our paper include data from other, countries raising a potential issue of generalizability to the UK.

The strengths of the paper are that it used four different databases in the search, and has included articles researching different specialities and countries. Furthermore, the review focused on HSE management standards, which is a new way to view this topic. HSE management standards influence risk factors for suicidal ideation in physicians. Despite this, there is a lack of research into this pertinent area.

The negative impact upon doctors’ health is already noted. There are huge economic and financial reasons as to why stress at work ought to be managed. It is, however, important to note the earlier finding from our literature search that doctors appear to require different types of support at different stages of their careers. As such, any intervention will need to be flexible and able to be personalised to combat an area as multifactorial and nebulous as stress management.

### 5.2 Role of Technology

A simple solution could be using an interactive mobile‐based application with a modified HSE standards framework to assist doctors to become self‐aware of changes in stress. During the literature search, we became aware of several web‐based self‐monitoring tools for physicians [51, 52]. These were not directly linked to the focus of this study, nor was there any significant scientific literature on them. In addition, the resident doctors of Canada (RDoC) has created a resiliency curriculum in conjunction with the Canadian forces and the mental health commission of Canada [53]. The curriculum contains a sliding scale called the mental health continuum, which measures the impact of stress upon the physician's life [53]. This gives the physician a visual representation of their stress from red‐green and allows them to self‐monitor [53]. Moreover, a well‐being app was proposed in a letter to the British journal of psychiatry, which draws upon salutogenesis [54, 55, 56]. This process encourages staff to focus on things that are ‘working well’ and utilise general resistance resources, rather than the negatives in the NHS workplace. Research has shown that a coaching intervention that encourages a salutogenic mind‐set results in the increase of well‐being and resilience of NHS staff [57].

We are also aware of several apps focussing on stress and mental well‐being for the general population. One such app is Youper, which not only monitors your mood and emotional well‐being, but also uses artificial intelligence to create a bespoke course of action, using methods such as mindfulness, meditation, cognitive behavioural therapy, and Acceptance and commitment therapy [58, 59].

With the HSE management standards in mind, the idea of a stress management and well‐being app for physicians has been devised (Supplementary information 3 and 4). We acknowledge that there can of course, be negative connotations when it comes to the use of such interventions, such as a progressive move away from human contact, privacy, and security concerns, as well as increased screen time usage that can result in added depression and sleep problems [60]. However, the aim of our app is to provide physicians with a platform to monitor their stress levels in relation to the HSE management standards. These levels can be recorded at frequencies dictated by the physicians, providing a clear visual representation of stress levels using a traffic light system. Self‐management advice is provided based on the outcome, and changes can then be monitored over time. This app will contribute to an increased self‐awareness of stress for physicians and the individual factors contributing to it. The well‐being app proposed is different from the other monitoring tools as it is easily accessible, quick to complete and self‐empowering. Moreover, we aspire that it can be used as a tool to open up conversations between doctors and their supervisors, providing them with a platform to validate their need for further support. However, this would require further research to understand the population this could be of benefit to and the potential for any harm it may cause. A full comparison can be found in Table 2.

**TABLE 2:** Comparison of web and App based self‐monitoring tools for physicians.

	Physician well‐being index [37, 38, 51]	Suicide prevention and depression awareness programme [52]	Youper [58, 59]	Proposed well‐being‐app
Purpose	Well‐being—Risk assessment	Depression and suicidal ideation—screening tool	Emotional well‐being Self‐awareness	Stress—self‐awareness
Number of questions	7	>10	Variable	6
Type of questions	Dichotomous	Multiple	Multiple	Likert
Physician can pose questions	No	Yes—to counsellor	No	No
Outcomes	Instant comparison of welfare scores to a national cohort. Access to local and national resources. Identification of the increased risk of personal and professional consequences from current status.	Split into three levels Individual recommendations were given by the counsellor. The counsellor answers questions posed by physician. Invitations were extended for personal assessment based on the level.	Artificial Intelligence uses answers to create bespoke plans. Techniques offered include meditation, mindfulness, Cognitive Behavioural Therapy, Acceptance and Commitment Therapy. The use of validated scales to track symptoms.	Traffic light‐graded Outcomes recommended based on grade.
Validated	Yes (physicians/residents—Medical student version also available)	Yes	Yes	No
Repeat period	Monthly	One off	Set by user	Set by physician
Tracking over time	Yes	No	Yes	Yes
Platform	Web‐based	Web‐based	App‐based	App‐based
Access to data	Physician and institution	Physician and counsellor	Self‐management tool	Self‐management tool

### 5.3 Specific Challenges for the App Development

The focus of this paper has been reliant on user self‐awareness. However, the risk of placing too much responsibility on individual doctors could remove the focus and indeed the responsibility of responsible organisations. It is important that ‘self‐awareness’ is an addition to any ongoing institutional change and not an alternative to it. Future research should investigate how this app could integrate with workplace support mechanisms rather than acting as a standalone tool.

Similarly, there needs to be a strong work stream in any technology development to negotiate ethical considerations, including data privacy. In particular, screening needs to be in place to identify anyone who is more psychologically distressed than what the app is capable of supporting. These needs built‐in reviews at baseline and in future assessments. Where there is concern of a deteriorating situation, adequate signposting to suitable services, and indeed a link with human interface for rapid assessment of needs is warranted.

A further area to recognise is the increasing exposure of mobile technology and its impact on mental health, particularly of doctors. Social media platforms, while enabling to connect with others and share experiences, can also contribute to feelings of inadequacy and low self‐esteem.

The excessive use of smartphones can interfere with sleep, which is crucial for mental health [69]. Thus, due consideration should be given to the potential unintended harm/consequences of any app development in this sphere.

An important limitation in the area of app development is the marketing and awareness of the tool itself. This could be achieved through the use of a social media campaign, collaboration with training programs for doctors, and developing links with local hospitals. It is important this is done after empirical validation or preliminary user testing data.

### 5.4 Current Situation

Post our searches emergent evidence continues to highlight the same on‐going concerns of psychological distress in doctors [70, 71]. A 2023 comprehensive review of various studies of death by suicide of medical practitioners highlighted that a critical risk mitigating factor is the social support network [70]. In addition, risk factors included issues identified in our review including high workload and micro aggressions [70]. A 2024 systematic review of 39 studies from 20 countries of all published studies of doctor suicides till 2020 highlighted the need for continued efforts in research and prevention of doctor deaths by suicide [71].

The recent Covid‐19 pandemic too could have put additional strain on the mental health of physicians, potentially exacerbating the recognised risk factors [70]. Looking specifically at medical trainee burnout, the GMC national training survey in April 2021 highlighted a third reported they felt burnt out to a high or very high degree because of their work, a higher proportion than in pre‐pandemic years, which increased to nearly two‐thirds (62%) a year later [72]. A cross‐sectional survey done on 424 UK trainee doctors between March 2020 and January 2021 assessed working conditions using HSE standards and its relationship to psychological symptoms generally and suicidal ideation particularly [73]. Of all respondents, 50.2% reported suicidal ideation [73]. Depression symptoms mediated all six HSE standard relationships though no direct link was found to suicidal ideation [73]. Further inquiry and mitigation of the indirect relationship between working conditions and suicidal ideation via depressive symptoms is needed [73]. Further, much of the research is the United States, Australasia and Europe with a noted lack of scarcity of research from many other areas such as Africa and Latin America [71].

## 6 Conclusion

Suicidal behaviour and severe stress in physicians has been shown to be polymorphic and multifactorial, and has numerous risk factors. It is thus important to have broad domains of risk and to ensure people measure their well‐being and stress against themselves. Of note are work‐related risks that have been identified which are linked to the HSE Management Standards. These give an indication that support focussed on factors pertaining to the individual physician alone is not enough to combat the rising concern of suicide in the profession. We propose a self‐monitoring and self‐empowering Stress management and wellbeing App as an innovative resource for physicians to monitor their stress levels to keep safe and recognise psychological threats to their well‐being.

## Author Contributions

**Kirsten Leslie**: conceptualization, data curation, formal analysis, investigation, methodology, validation, visualization, writing – original draft. **Chloe Sawyer**: conceptualization, data curation, formal analysis, investigation, methodology, validation, visualization, writing – original draft. **Katy Oak**: investigation, methodology, project administration, resources, visualization, writing – review and editing. **Gareth Lewis**: investigation, project administration, resources, software, validation, visualization, writing – review and editing. **Bryan Clark**: investigation, project administration, resources, software, validation, visualization, writing – review and editing. **Anna Mankee‐Williams**: investigation, methodology, visualization, writing – review and editing. **Ellen Wilkinson**: investigation, methodology, visualization, writing – review and editing. **Hiu Lam**: investigation, methodology, visualization, writing – review and editing. **Richard Laugharne**: investigation, methodology, visualization, writing – review and editing. **Rohit Shankar**: conceptualization, data curation, formal analysis, funding acquisition, investigation, methodology, project administration, resources, supervision, validation, visualization, writing – review and editing.

## Conflicts of Interest

RS has received institutional and research support from LivaNova, UCB, Eisai, Neuraxpharm, Bial, Angelini, UnEEG and Jazz/GW pharma outside the submitted work. No other author has any declared conflict of interest related to this paper.

## Supporting information



Supporting Information 1

Supporting Information 2

Supporting Information 3

Supporting Information 4

## Data Availability

All data used for the paper is within the manuscript.

## Appendix A Search strategy

### A.1

Medline HDAS:

**Table:**

1	(Doctor OR doctors OR physician OR physicians). ti, ab
2	Physicians/
3	(1 or 2)
4	Suicide/ OR ‘suicidal ideation’/ OR ‘suicide, attempted’/
5	‘Self‐injurious behavior’/
6	(suicid* OR self‐harm OR self‐injur*). ti, ab
7	(4 OR 5 OR 6)
8	(3 and 7)
