# Supplementary material for: A Self‐Monitoring Mobile App to Mitigate Risk Factors for Suicide and Self‐Harm in Junior (Resident) Doctors: A Review, Thematic Analysis and Concept Proposal
Source: Healthc Technol Lett. 2025 May 6;12(1):e70009. doi: 10.1049/htl2.70009 (PMC12054714; doi:10.1049/htl2.70009)
Supplement: Supplementary file 1 — Supporting Information 1 [file HTL2-12-e70009-s002.docx]

| **HSE Management Standard** | **Composite Parts** |
| --- | --- |
| Demand | Workload, work environment, work patterns |
| Control | Autonomy over the way work is completed |
| Support | Encouragement, sponsorship and resources |
| Relationships | Promoting constructive working, managing unacceptable behaviour |
| Role | Understanding your part in the organisation, ensuring there is no role conflict |
| Change | Management of organisational change, communication of change |

**Supplementary Information 1**

Table 1 - HSE Management Standards – roles of the organisation to reduce stress. [1]

Reference

1. Health and Safety Executive. What are the Management Standards? Retrieved on 26 January 2019. <http://www.hse.gov.uk/stress/standards/index.htm>
