# Supplementary material for: A Self‐Monitoring Mobile App to Mitigate Risk Factors for Suicide and Self‐Harm in Junior (Resident) Doctors: A Review, Thematic Analysis and Concept Proposal
Source: Healthc Technol Lett. 2025 May 6;12(1):e70009. doi: 10.1049/htl2.70009 (PMC12054714; doi:10.1049/htl2.70009)
Supplement: Supplementary file 3 — Supporting Information 3 [file HTL2-12-e70009-s001.pdf]

2. EXISTING USER

Opening screen	User registration	Password / type or resend		Welcome page	Menu page

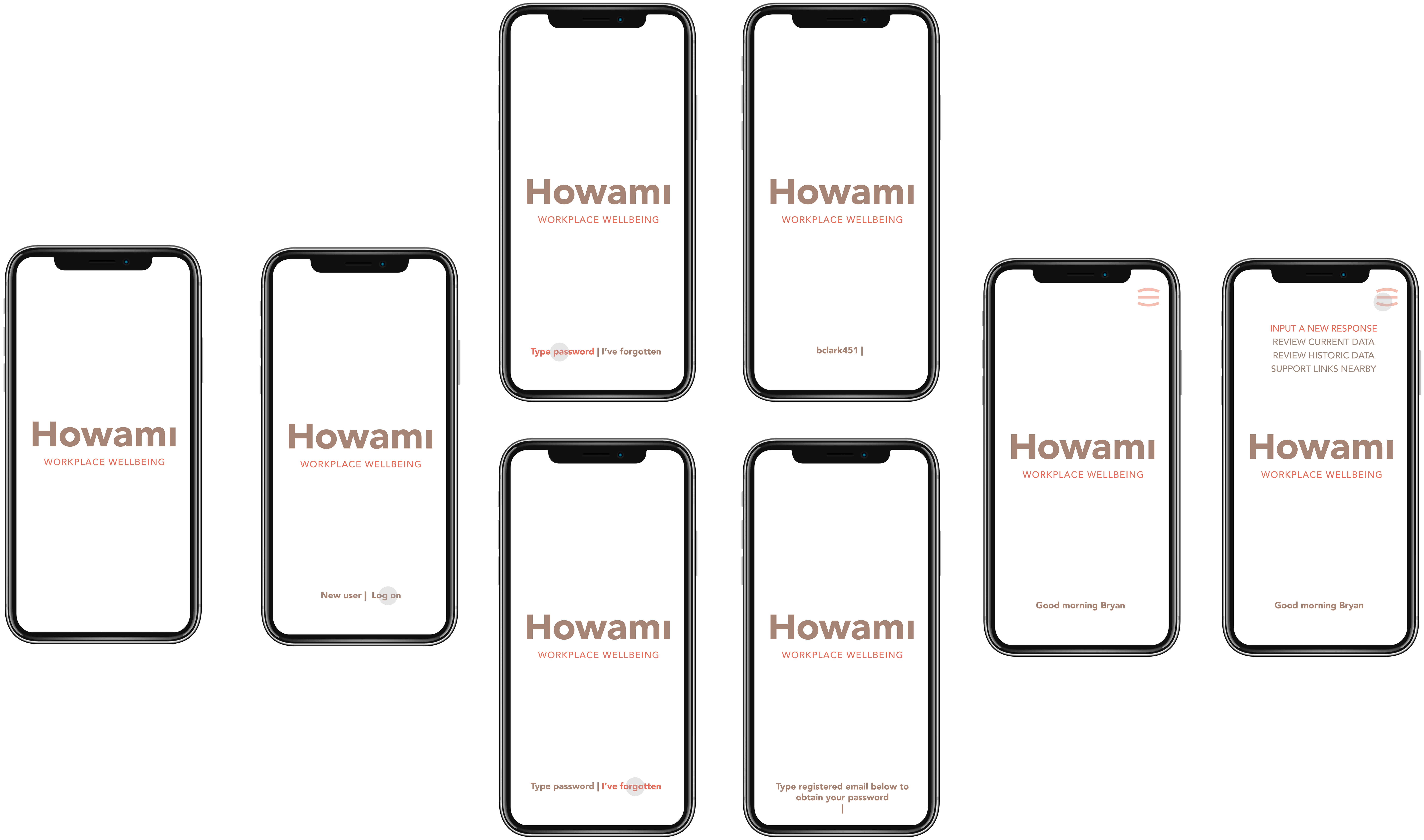

Description		Cursor flashes to prompt typing; correct code takes straight to next page or I've Forgotten	Existing user inputs code and forgotten passwords prompted to enter email for resend	Logged-in user can now select next step from menu.	Options provided for input and review
Effects / animation / sound		SFX; Thoughtful hmmmm sounds when input made			

3. TRANSITION TO QUESTION / DATA INPUT PAGE

Menu selection	Response 'smile' revealed selection	Transition page	Mouth revealed	Animation sequence	First question page

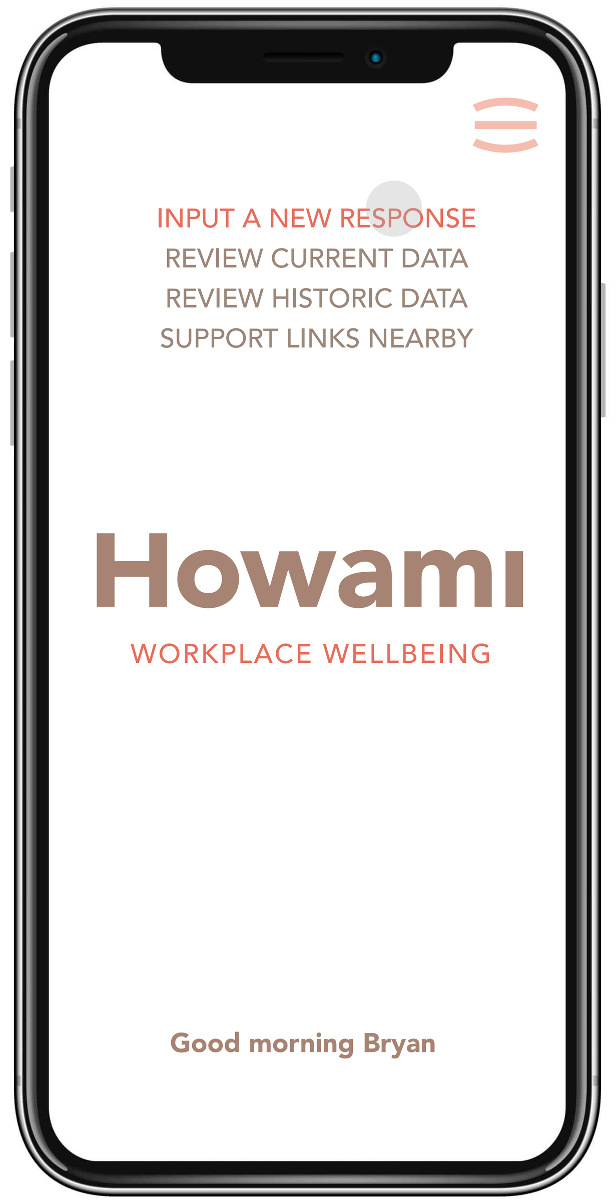

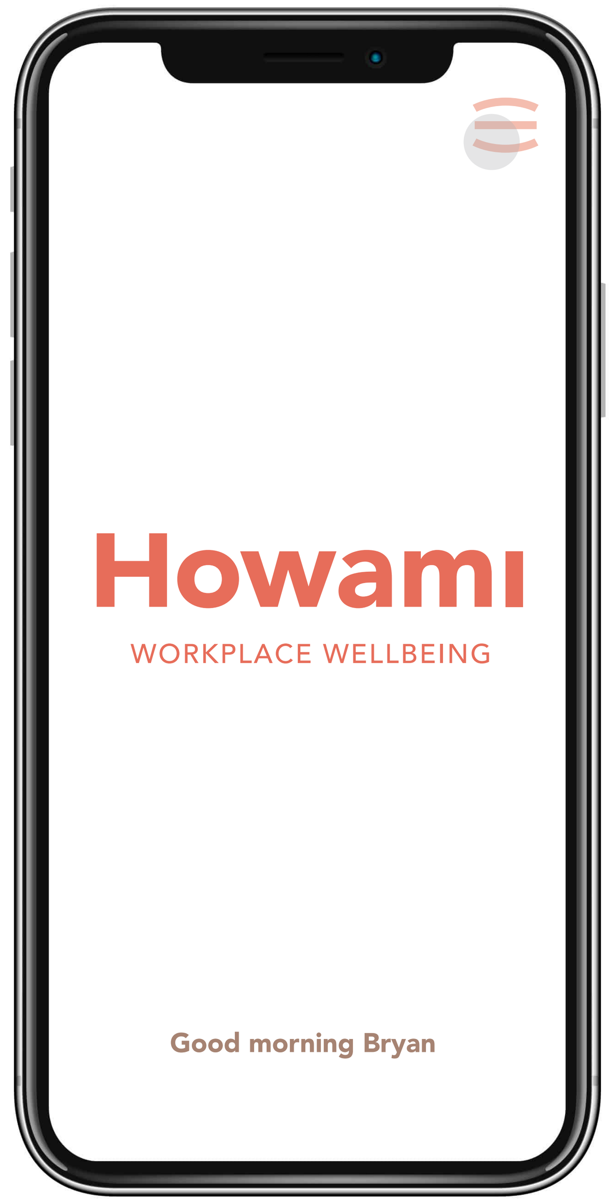

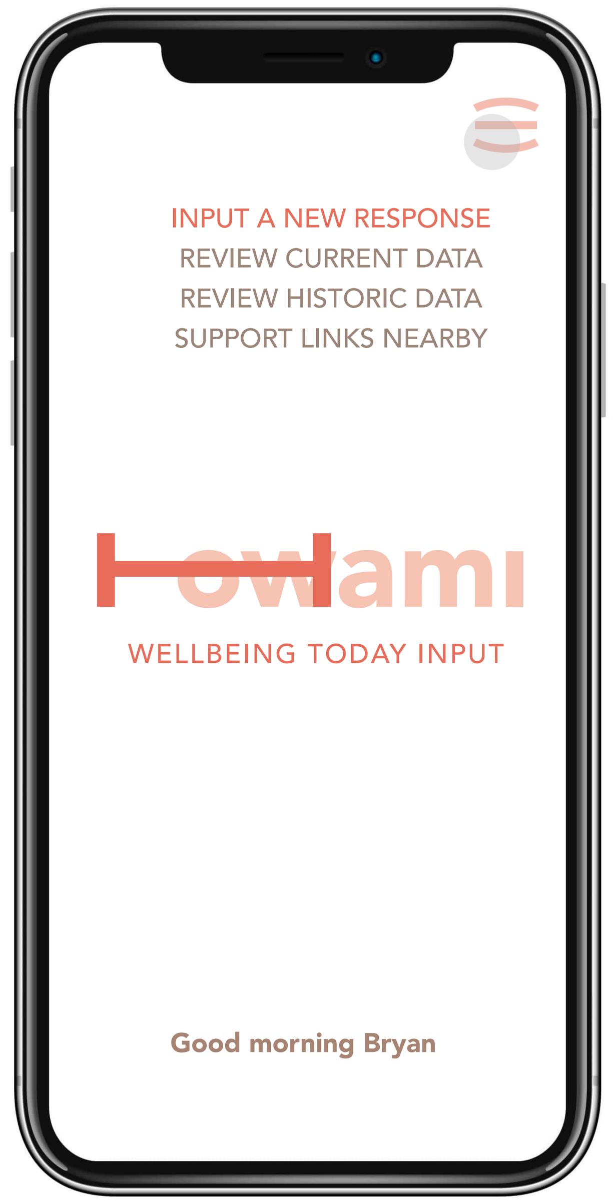

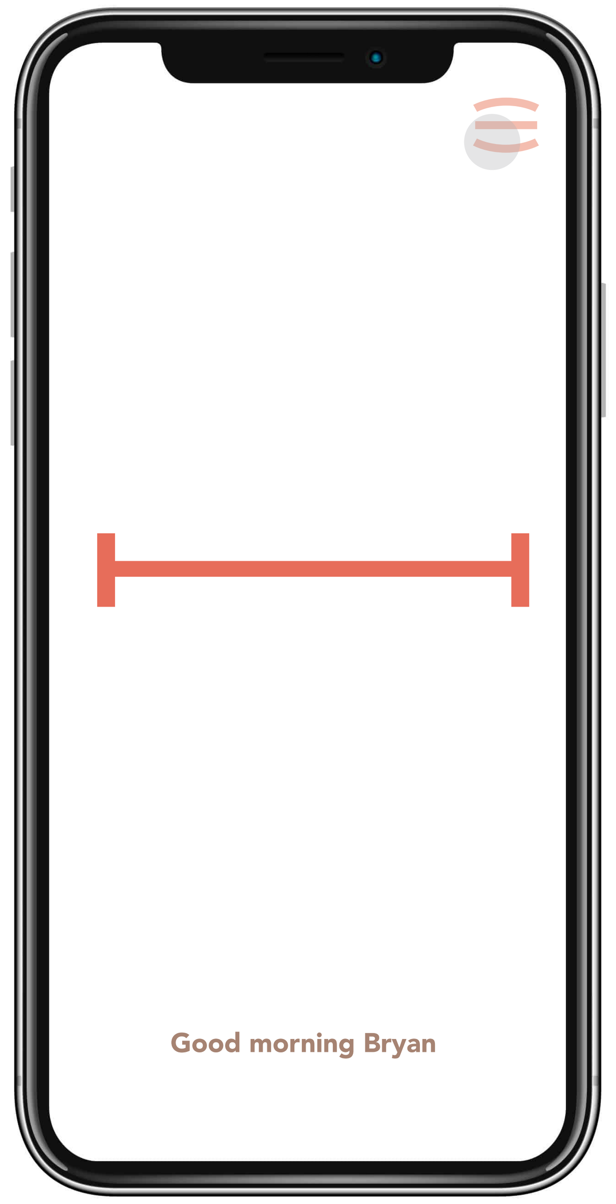

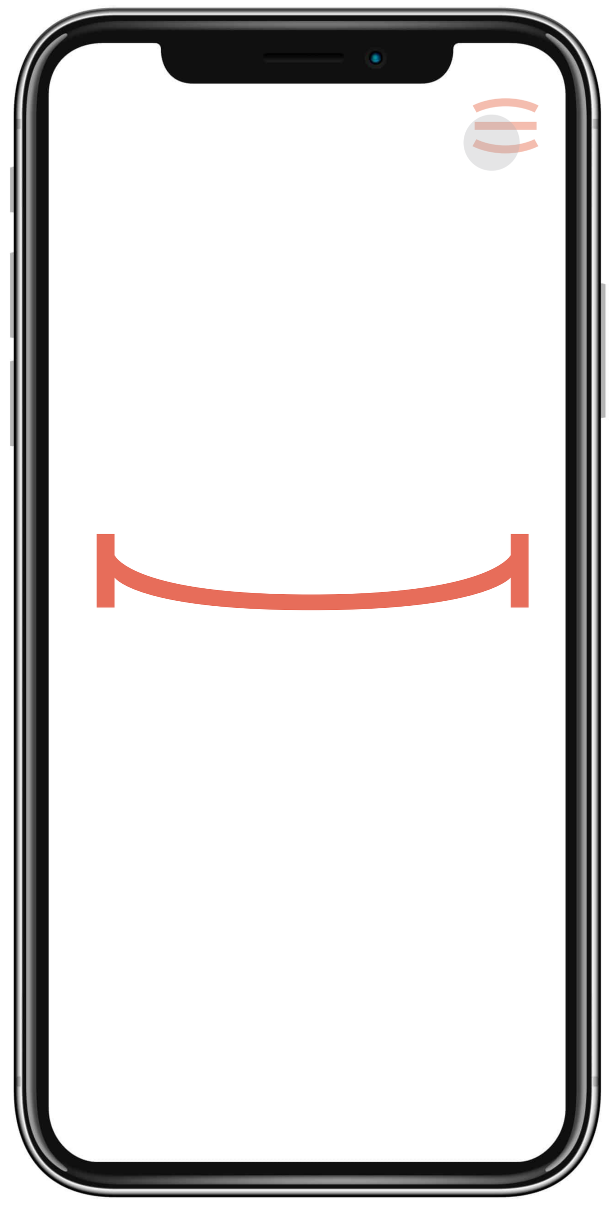

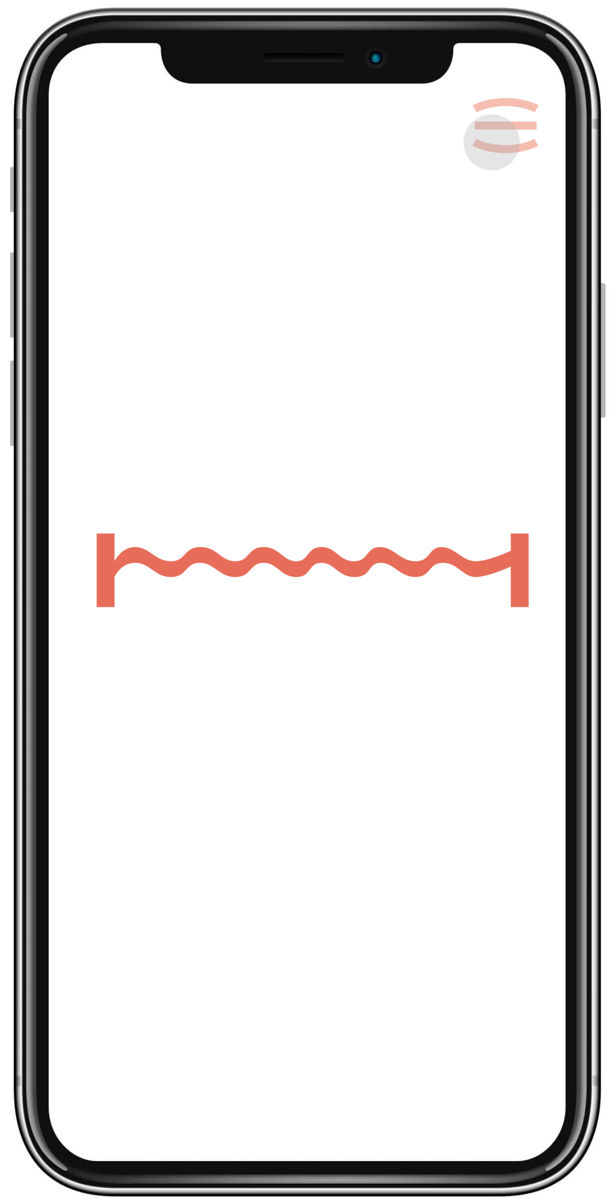

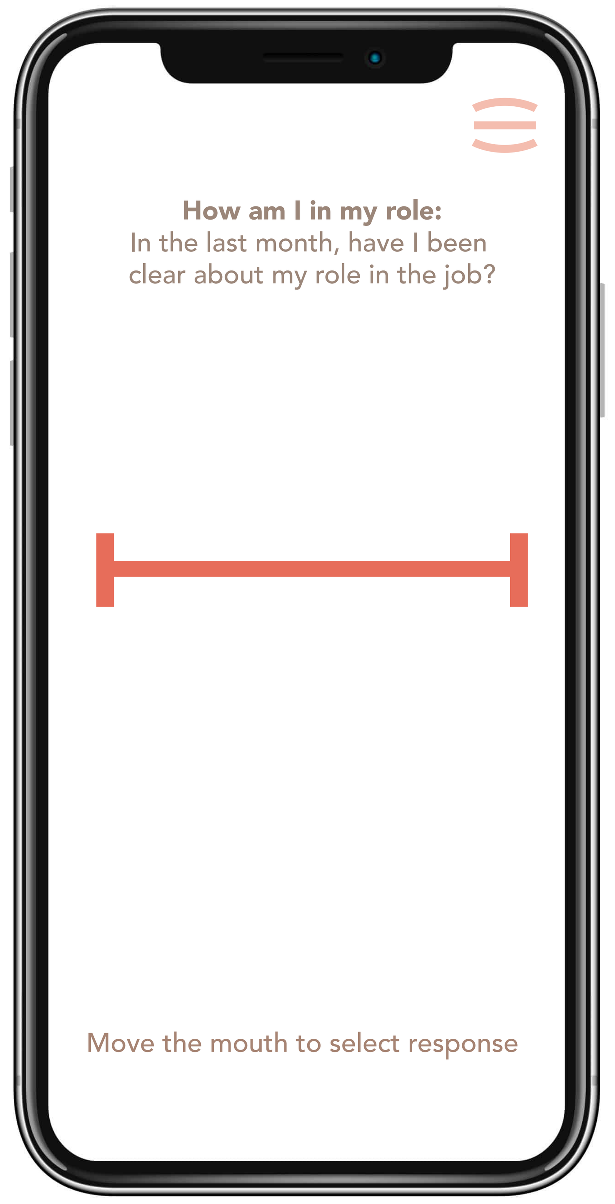

Description

Mouth device revealed across logotype

Existing user inputs code and forgotten passwords prompted to enter email for resend

Initial question revealed with prompt to select answer

Effects / animation / sound

SFX; Thoughtful hmmmm sounds when input made
Animation; 2 second movement of mouth across moods

4. QUESTION / ANSWER SELECTION

Question 1	Answer selected	Transition page	Mouth revealed	Answer page

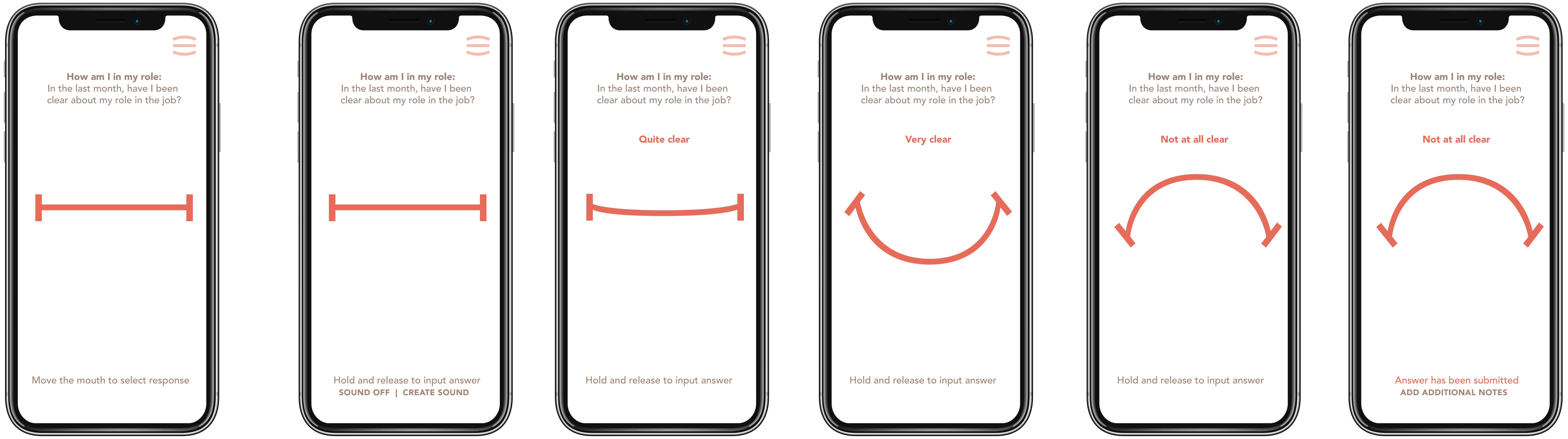

Mouth animation guide

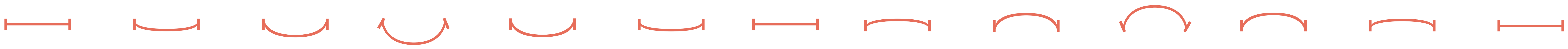

**Description**	User prompted to select answer<br>Option to turn sound on or create custom sound	User can scan through answers	Final Answer with option to record notes<br>via SIRI or keyboard
**Effects / animation / sound**		SFX; Thoughtful hmmmm sounds when input made or select from library or record own; positive / negative hmmms, expletives / more emotive (fu***** Sh**, Amazing), Animal sounds (dog growl, Cockerel crow), Effects (fart, fanfare)<br>Animation; Smooth transition of mouth to different states. Final selection to move slightly once selected.	

1. NEW USER REGISTRATION

Opening screen	User registration				Log-in page

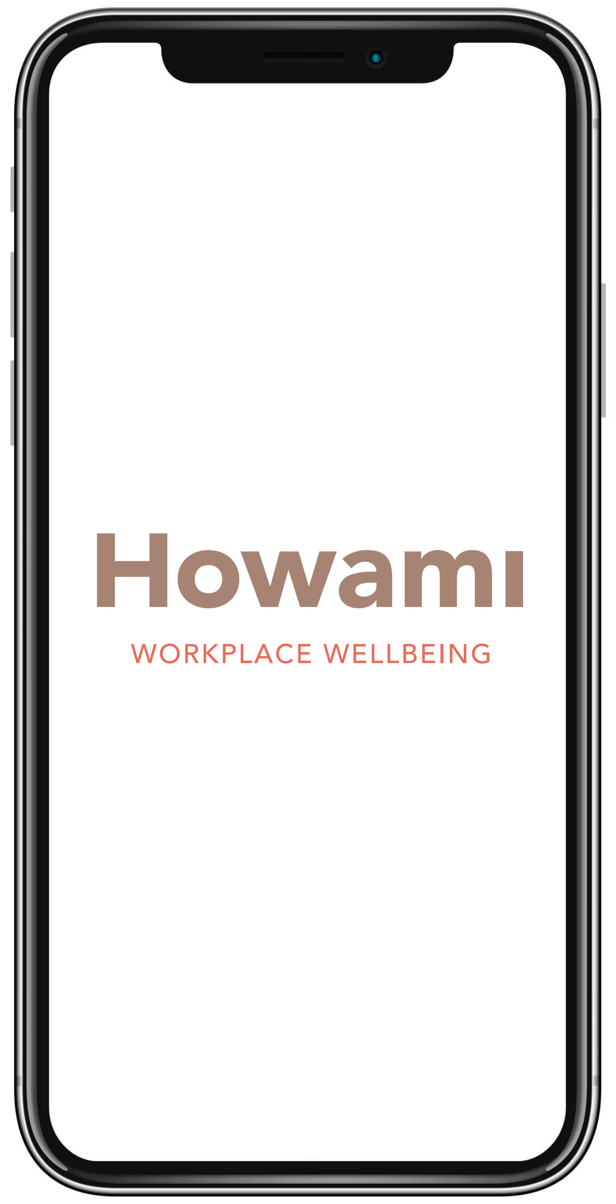

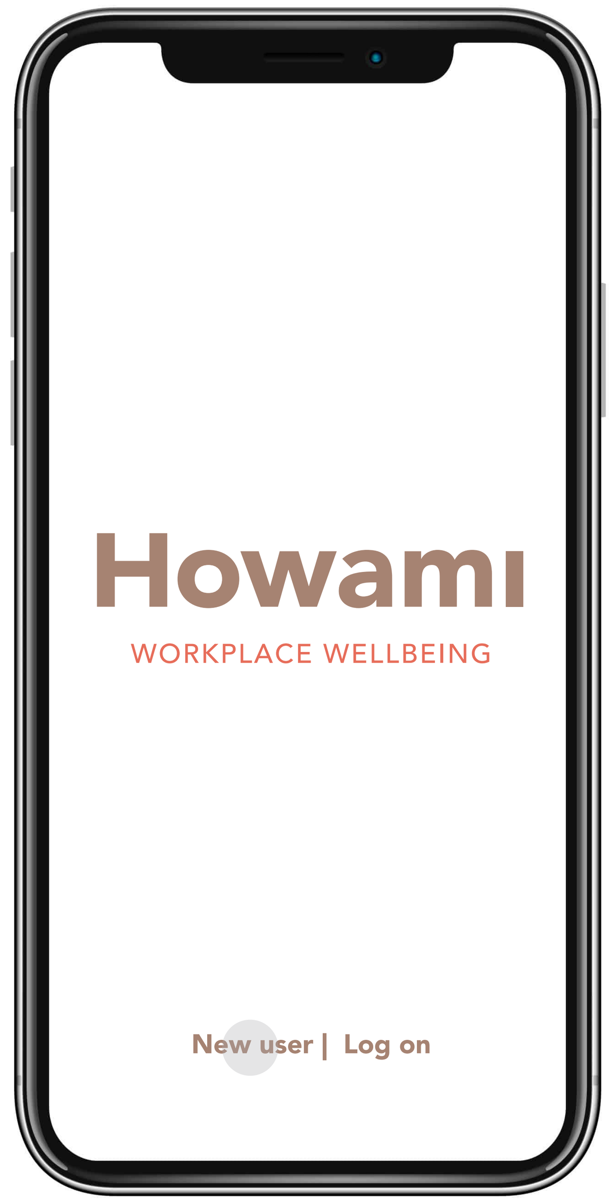

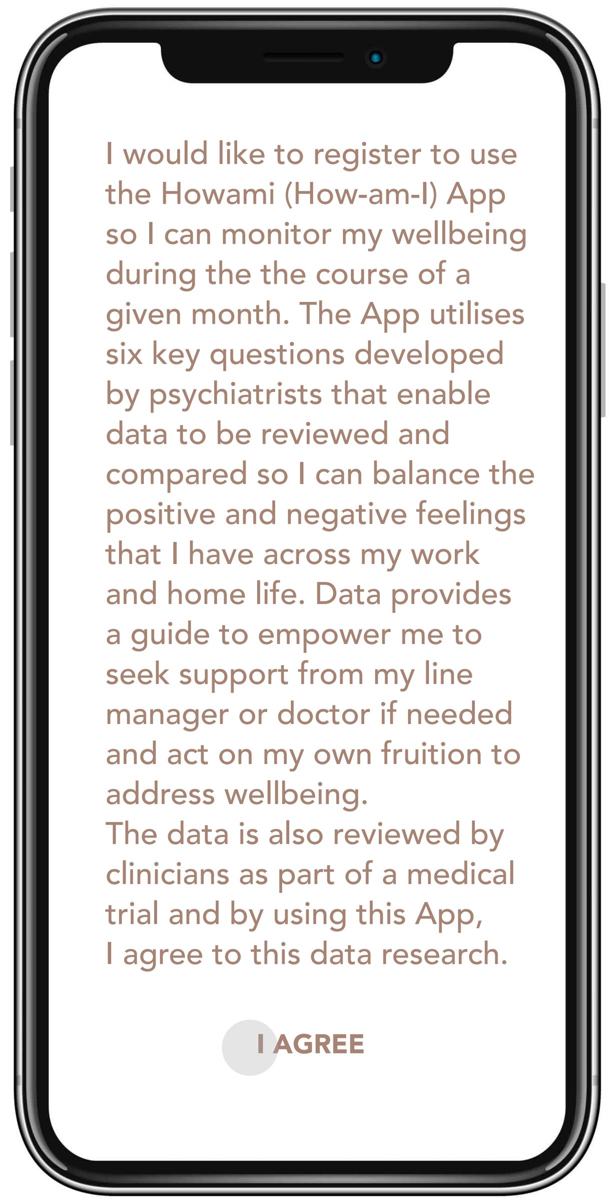

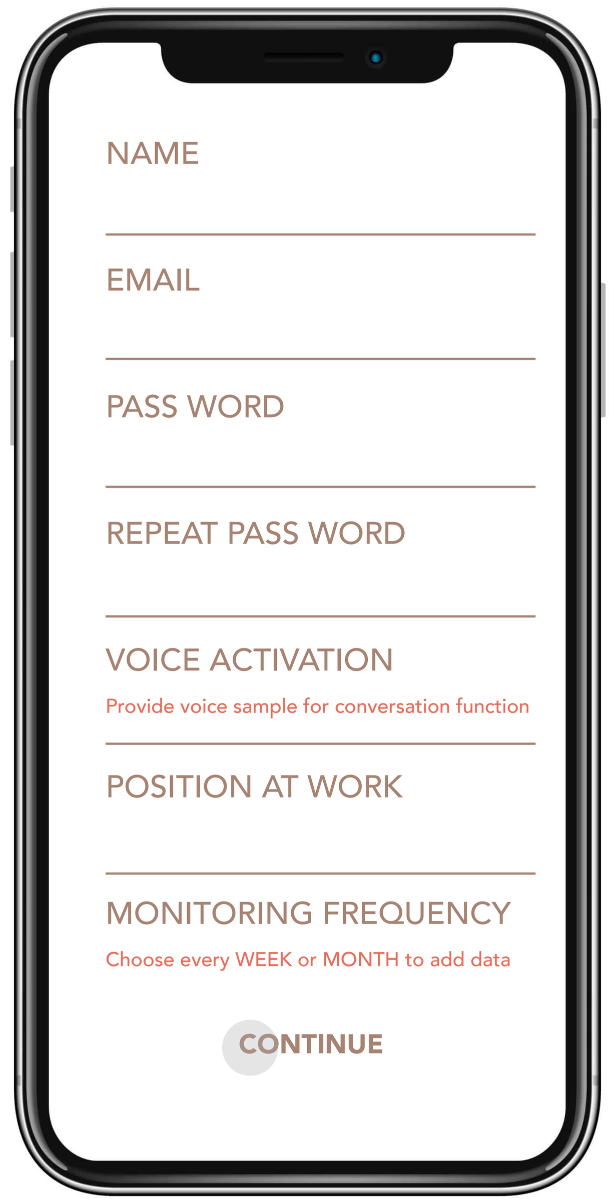

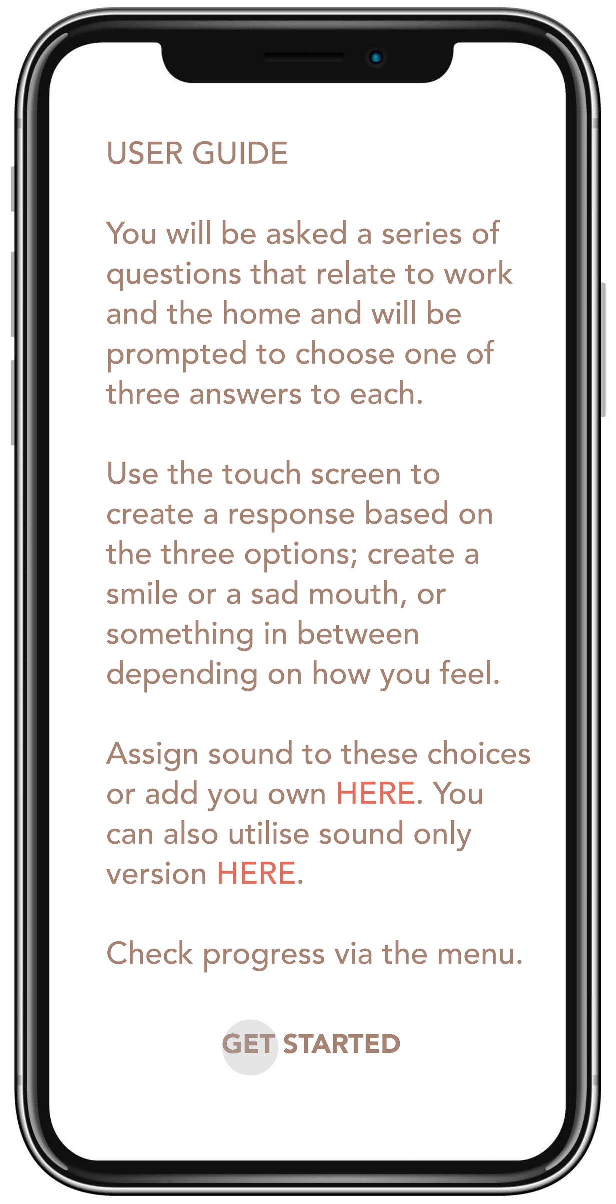

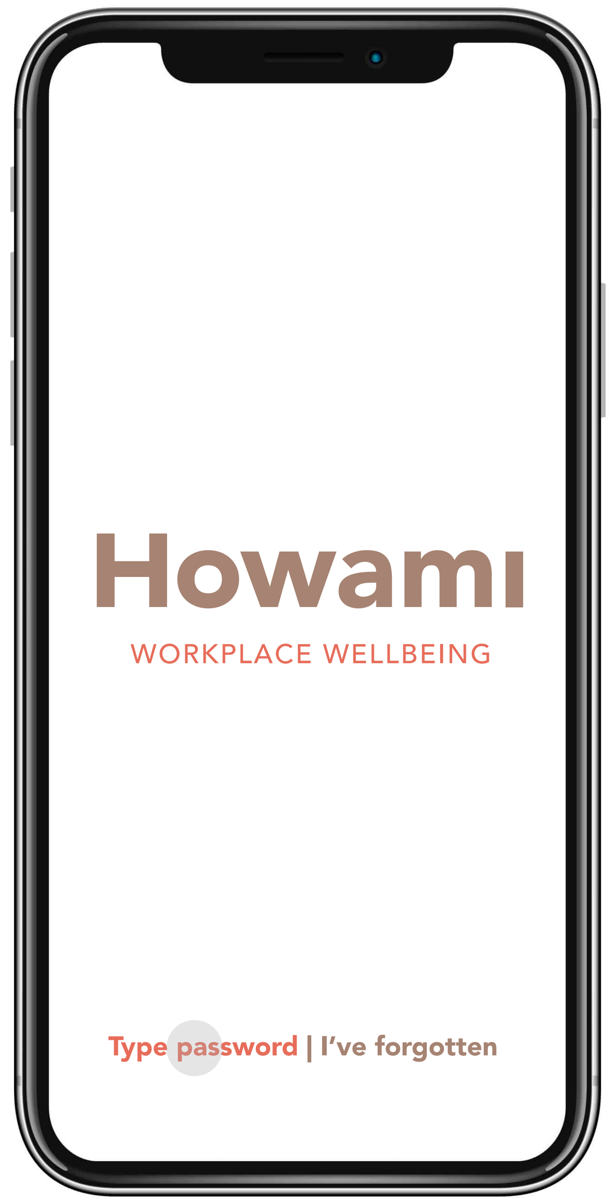

**Description**	**Cursor flashes to prompt typing; Selection takes straight to next page**	**New user taken to statement page, with 'I agree' button, prior to details**	**New user taken to registration page, with options on sound and frequency.**	**New user taken to instruction page, with options on sound creation and delivery**	**New user directed to log-in page as per a registered user**
**Effects / animation / sound**	**SFX; Thoughtful hmmmm sounds when input made**				
