# Supplementary material for: A Self‐Monitoring Mobile App to Mitigate Risk Factors for Suicide and Self‐Harm in Junior (Resident) Doctors: A Review, Thematic Analysis and Concept Proposal
Source: Healthc Technol Lett. 2025 May 6;12(1):e70009. doi: 10.1049/htl2.70009 (PMC12054714; doi:10.1049/htl2.70009)

5. QUESTION VARIANTS

Question 1	Question 2	Question 3	Question 4	Question 5	Question 6

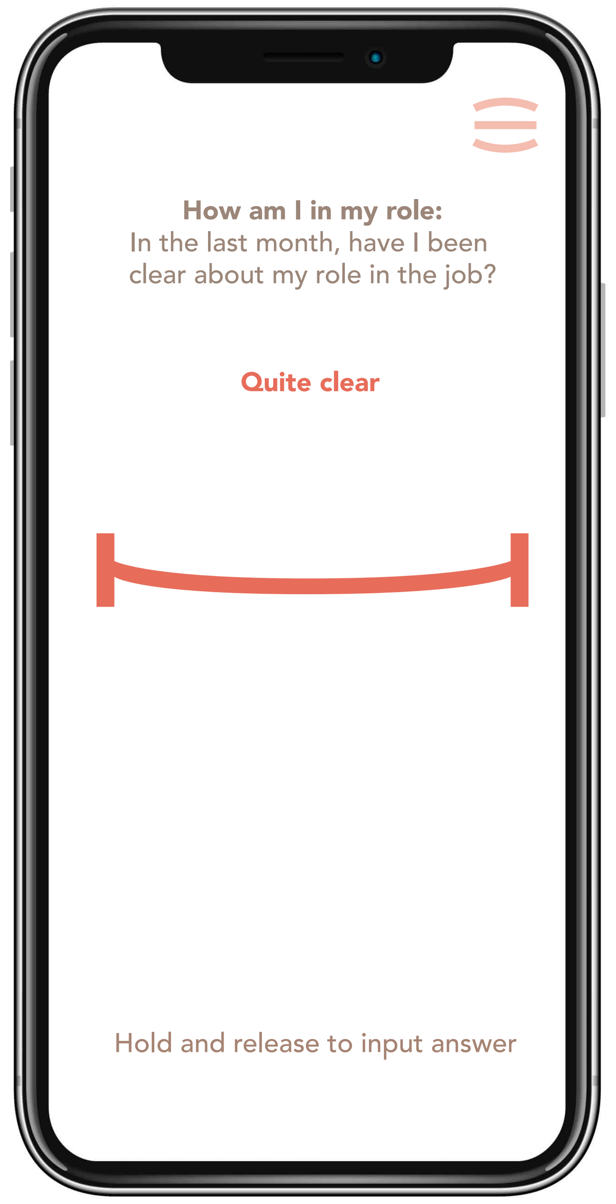

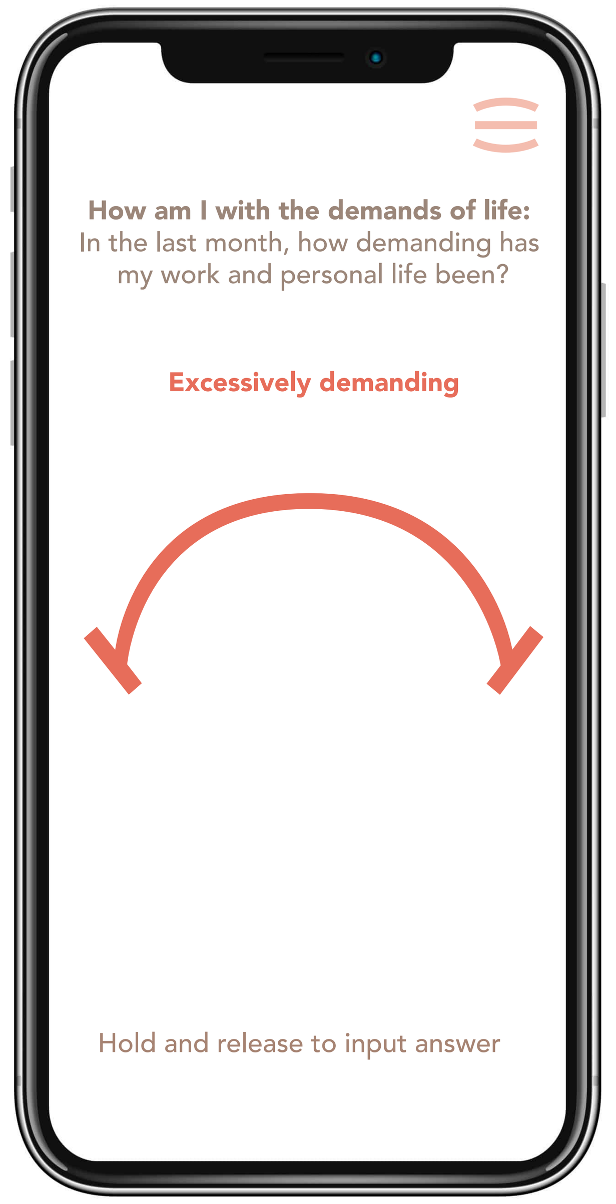

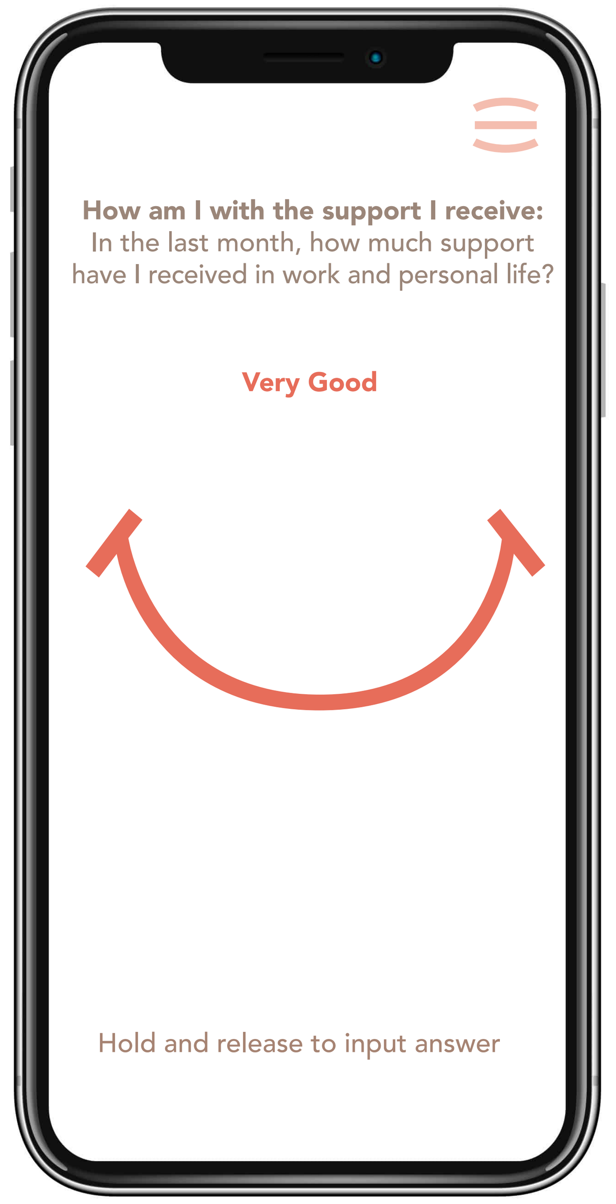

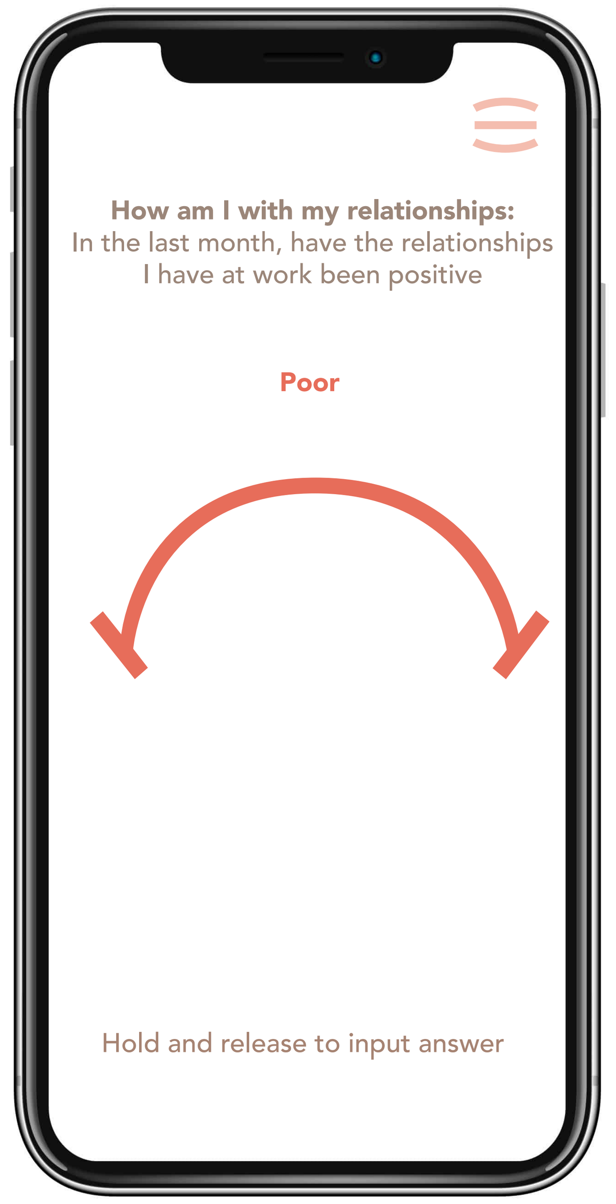

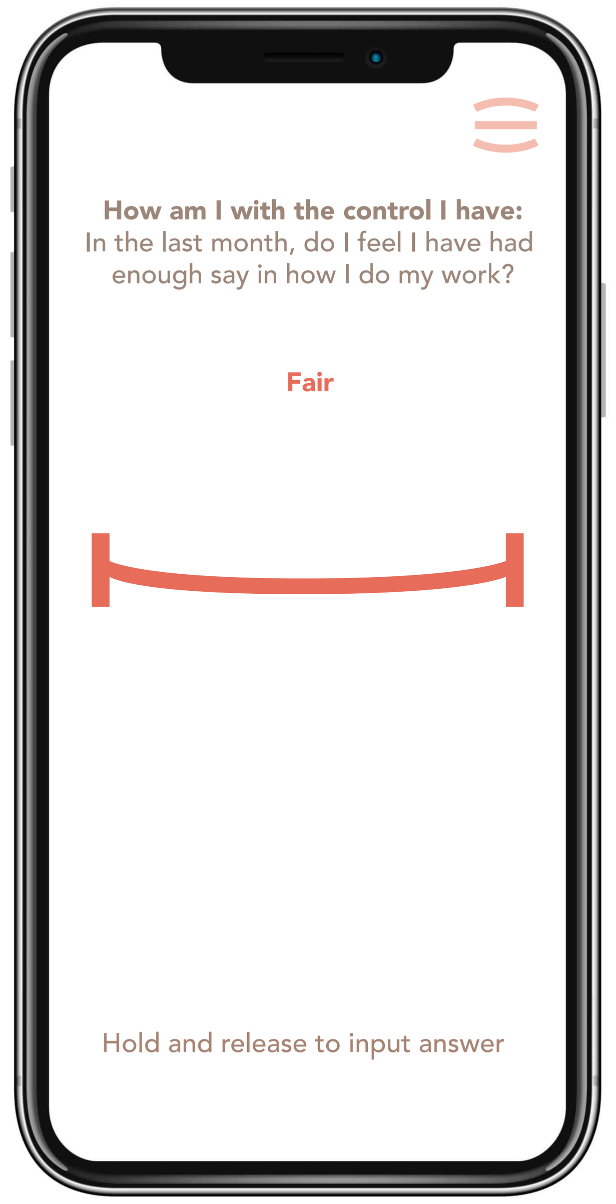

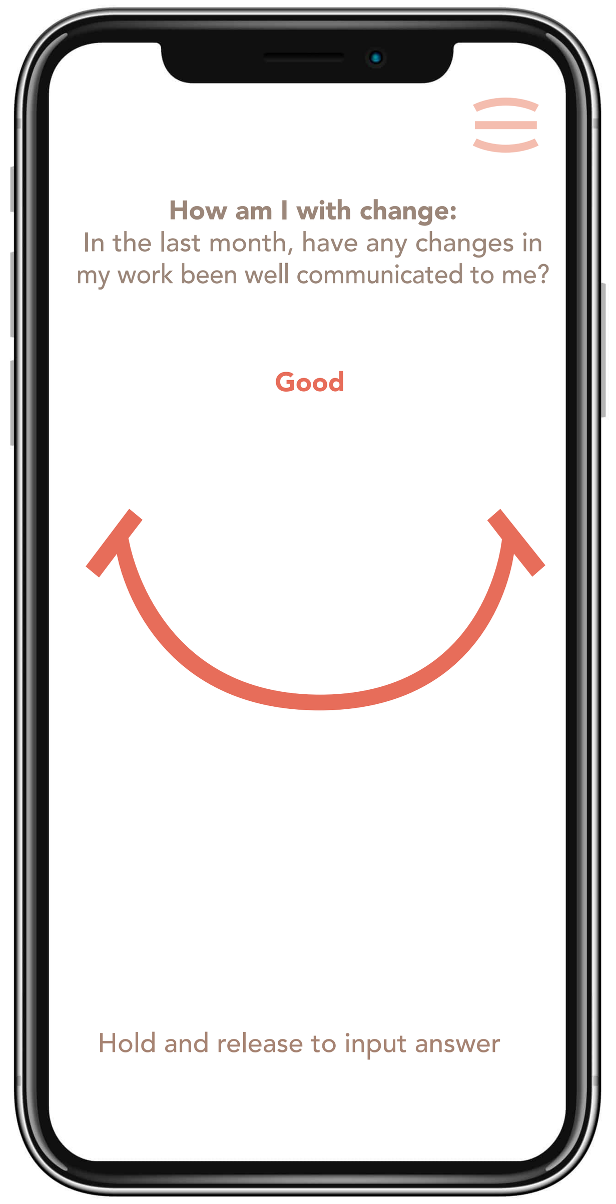

**Description**	**Additional questions utilise same functionality as page 4 detail**
**Effects / animation / sound**	**SFX; Thoughtful hmmmm sounds when input made or select from library or record own; positive / negative hmmms, expletives / more emotive (fu***** Sh**, Amazing), Animal sounds (dog growl, Cockerel crow), Effects (fart, fanfare)**<br>**Animation; Smooth transition of mouth to different states. Final selection to move slightly once selected.**

6. REVIEWING CURRENT DATA

Last question completion	Completion screen	Menu	Summary	Note recall page / Average result	Home page

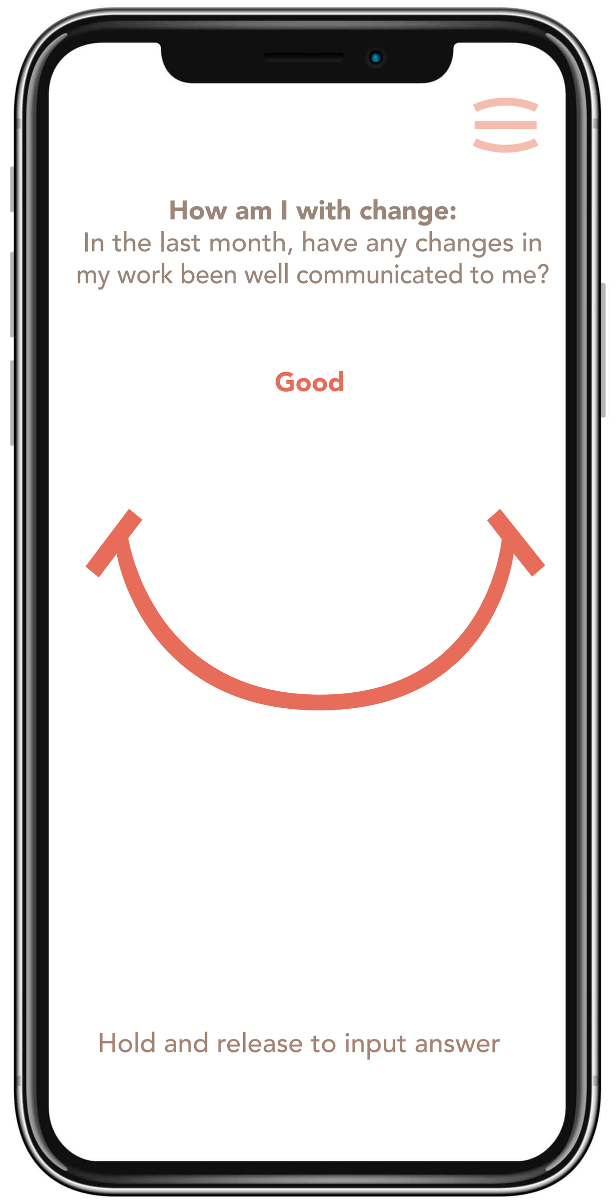

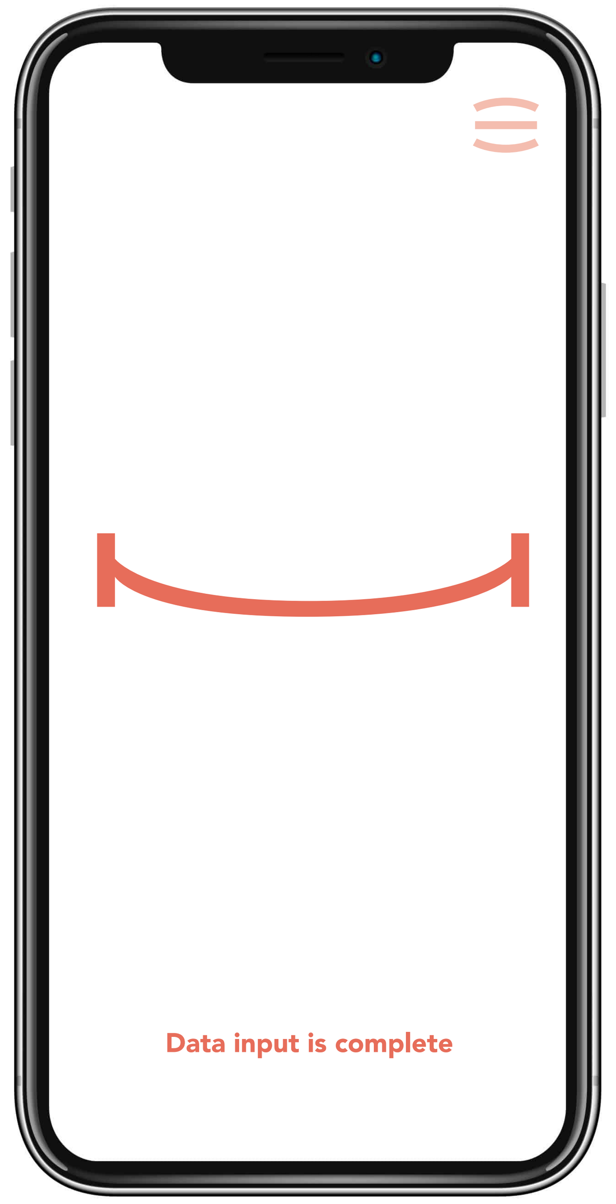

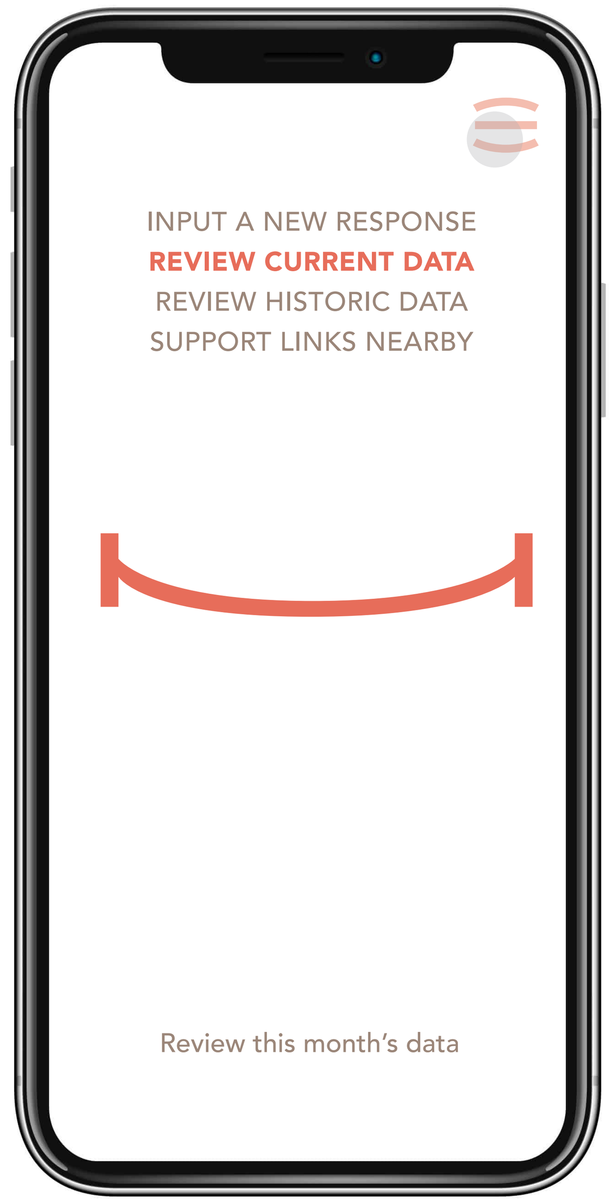

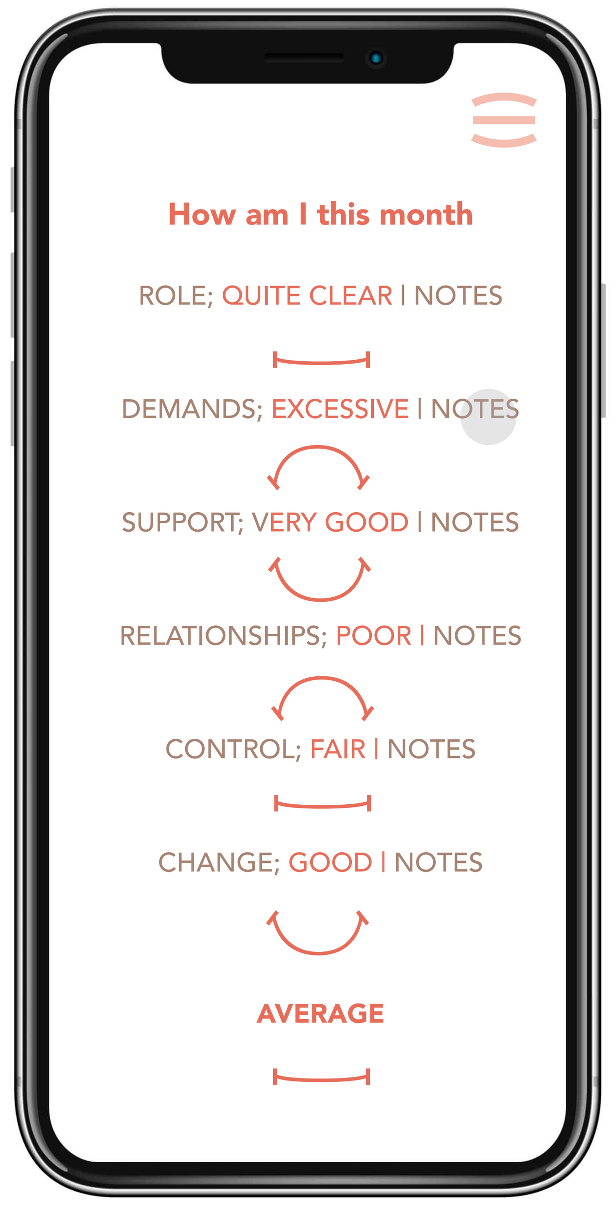

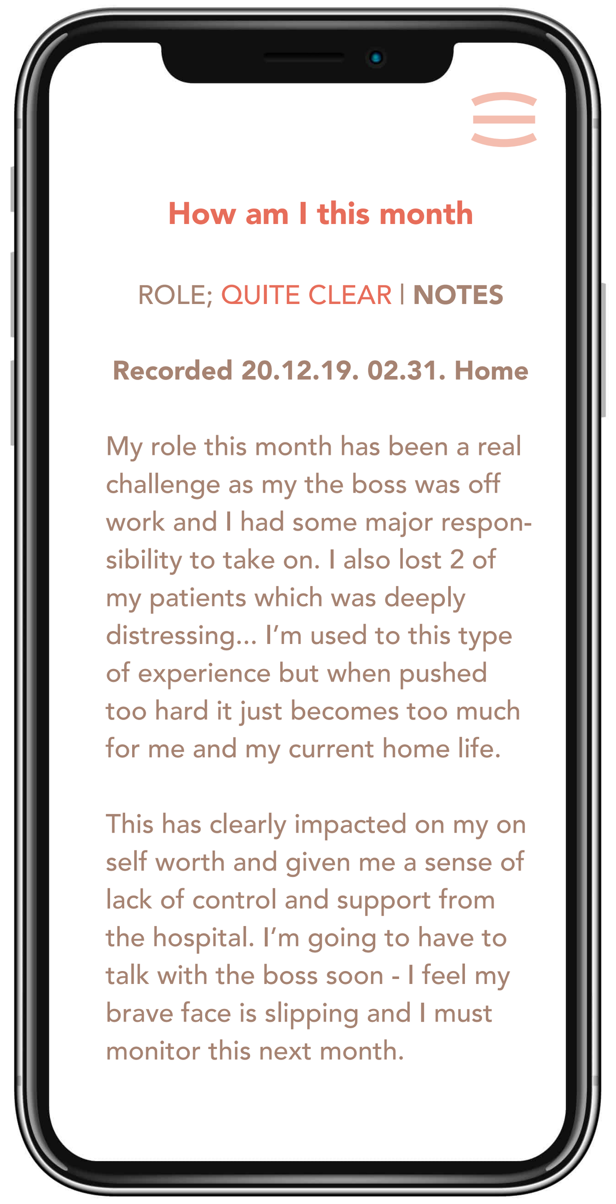

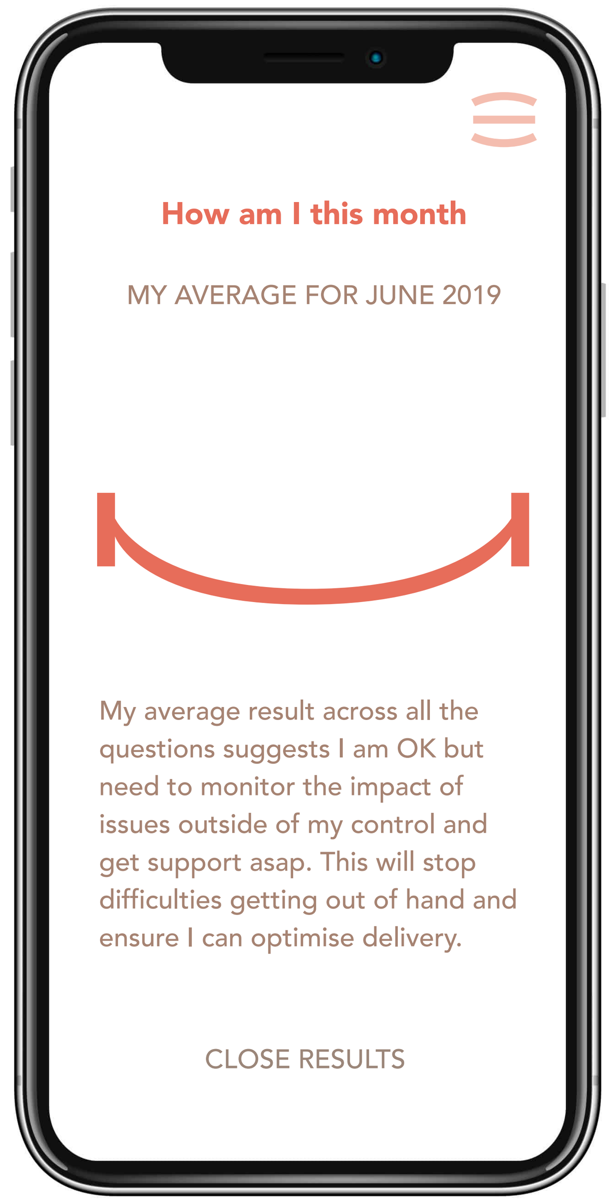

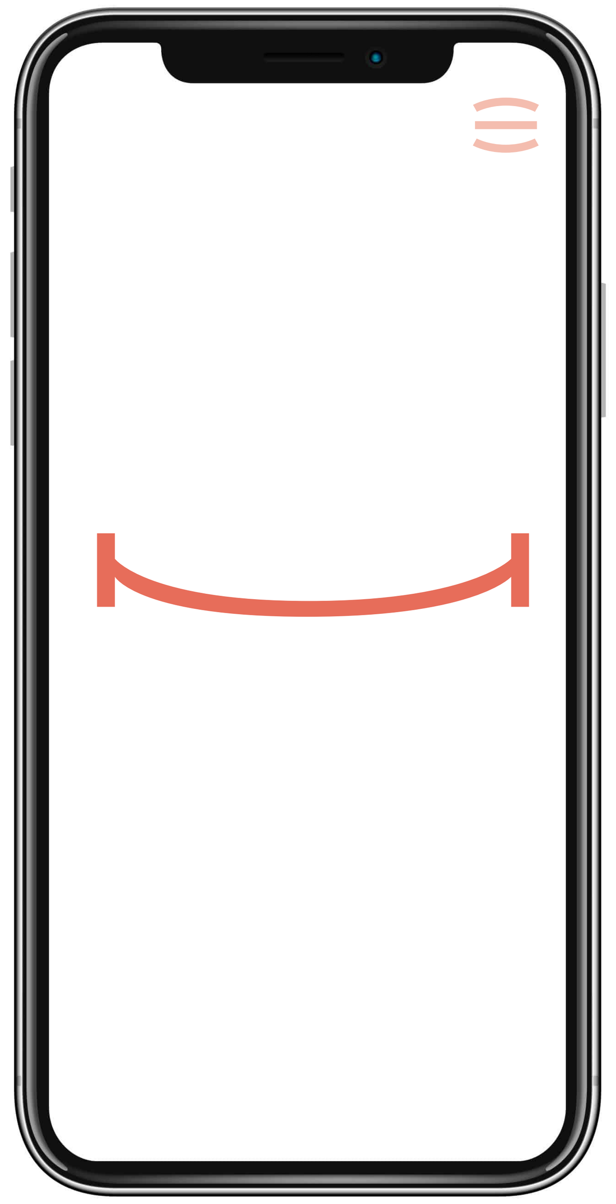

**Description**	**Input complete**	**Option to review data via screen prompt or main menu**	**Summary results including average response Clicking on 'Notes' allows individuals to link detail**	**Summary notes for single question or Average overview with recommendation**	**Screen saver mouth returns**
**Effects / animation / sound**	**SFX; Thoughtful hmmmm sounds Animation; Slight movement to mouth.**				

7. REVIEWING HISTORIC DATA / SUPPORT LINKS

Home page	Historical data selection	Historical results	Summary	Home page access to links	Links and support services

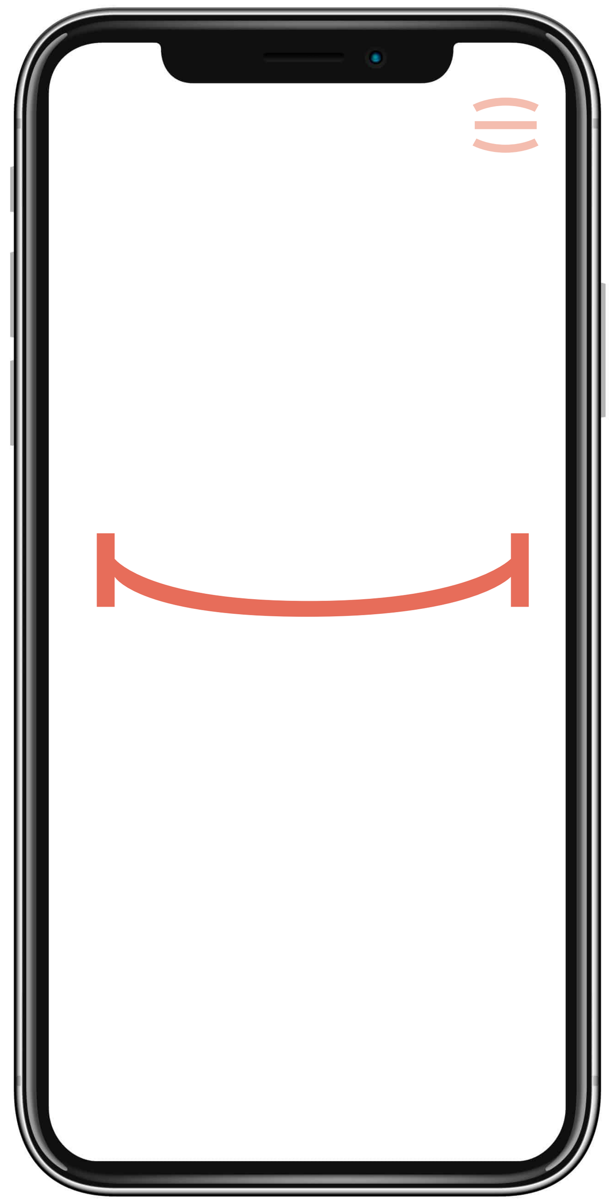

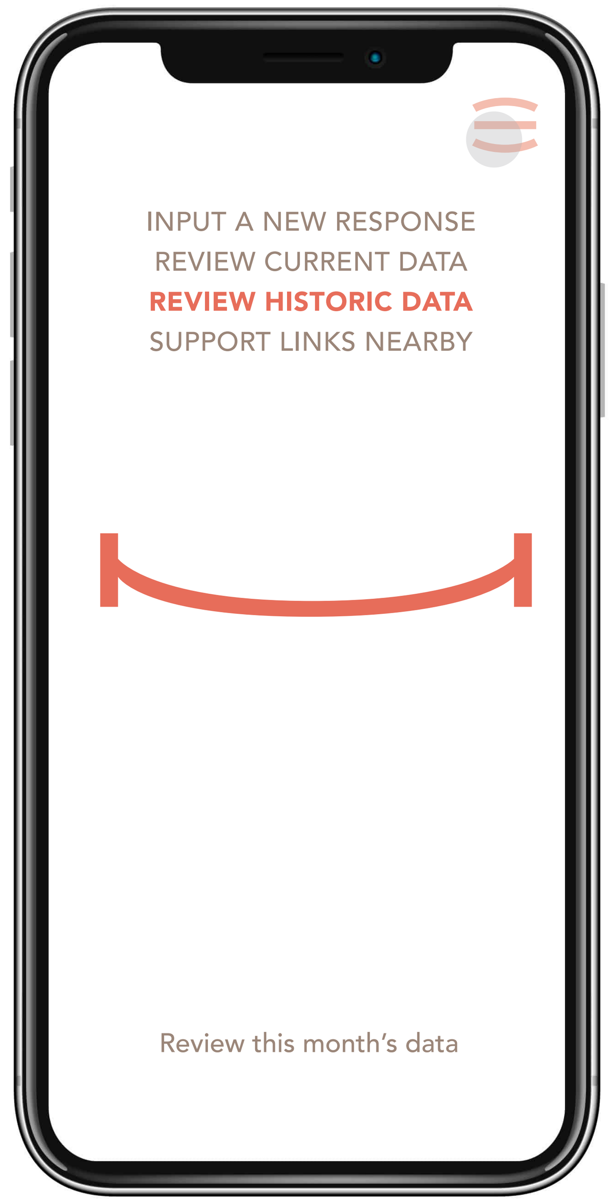

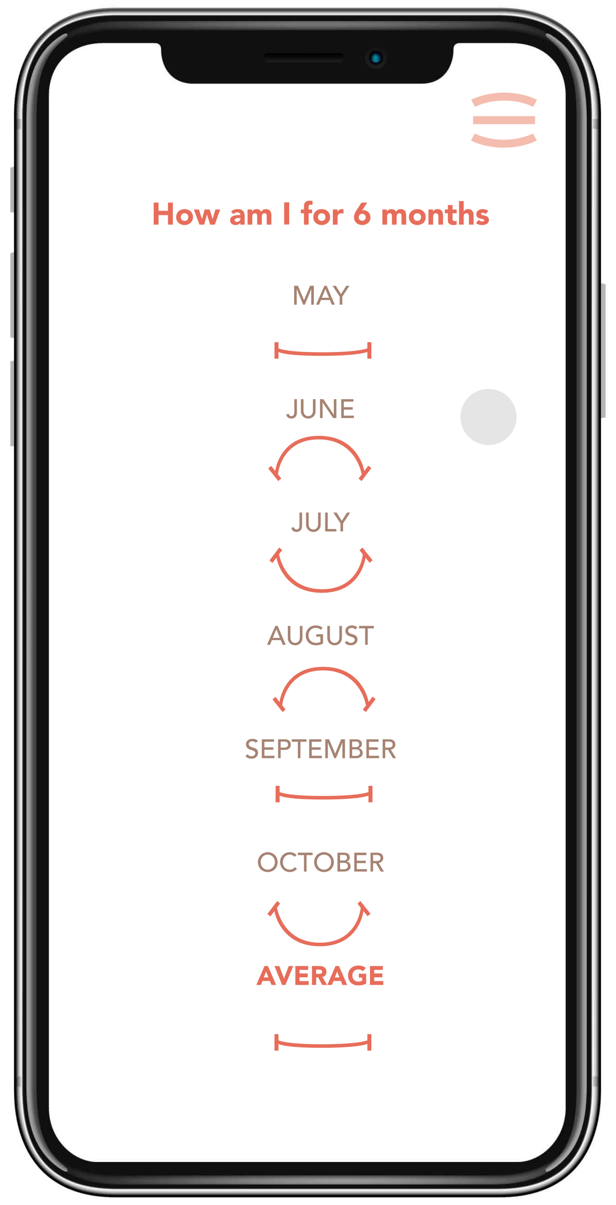

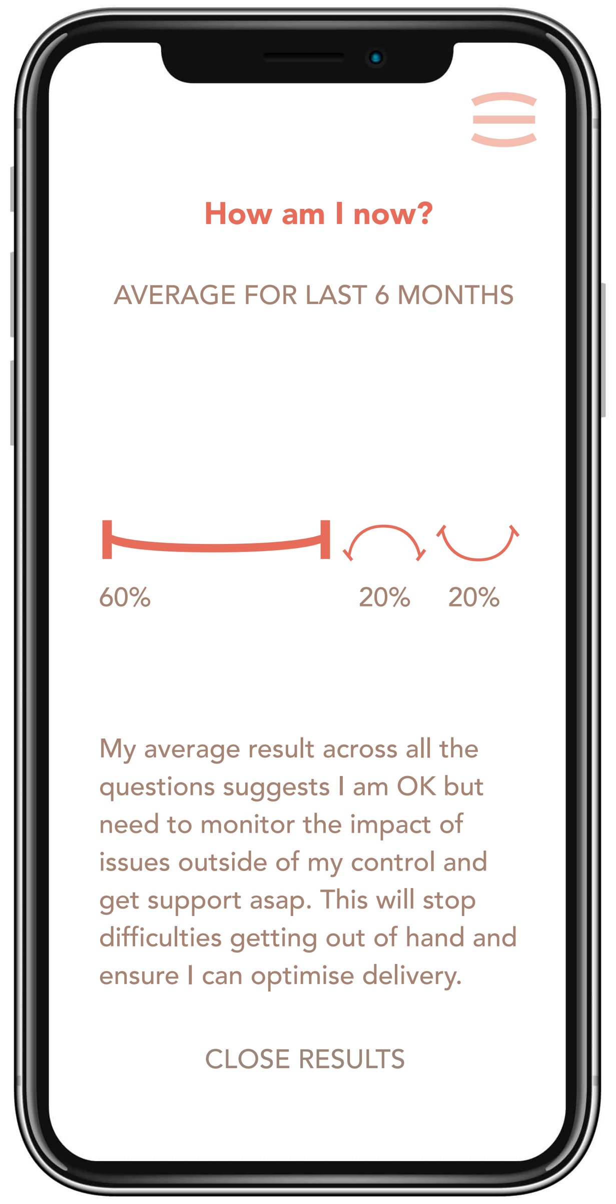

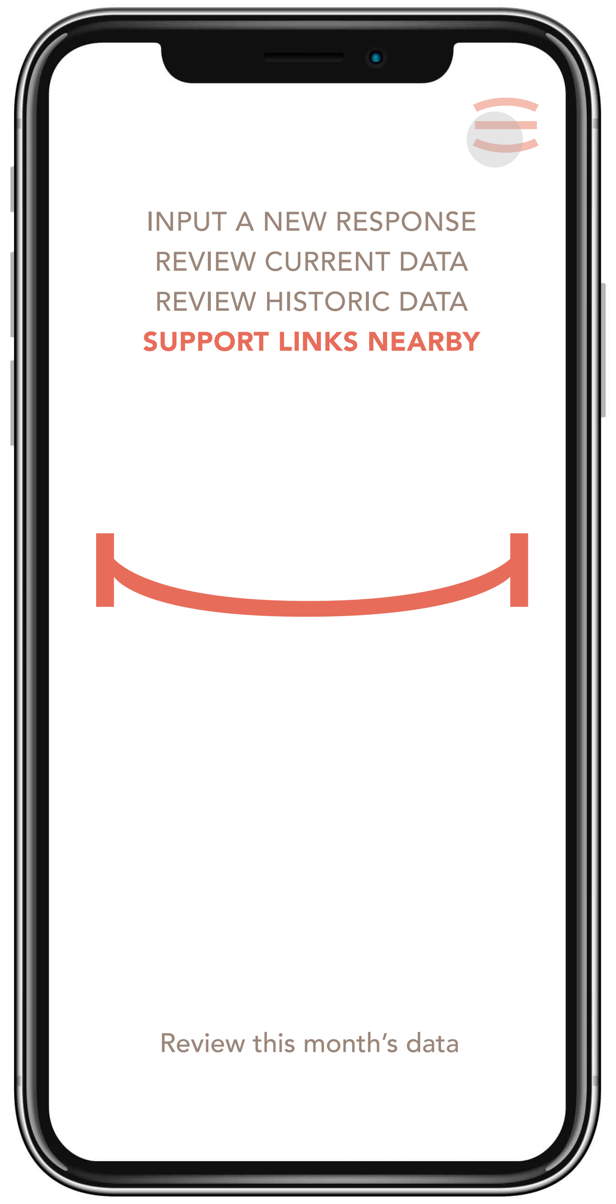

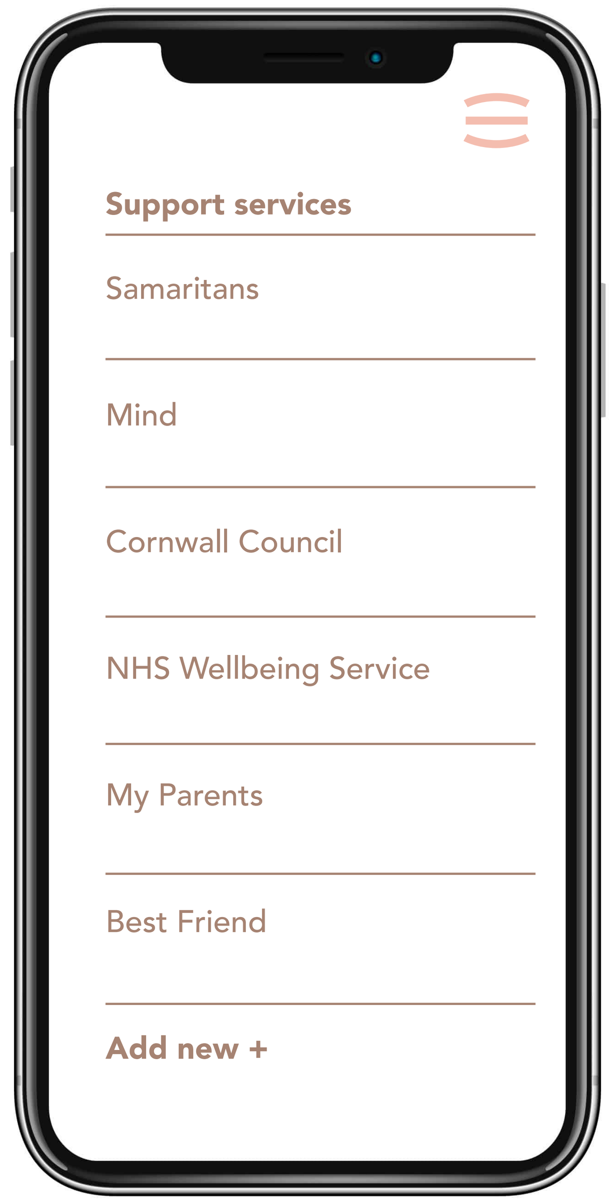

**Description**	**Input complete**	**6 Monthly summary used to generate average for overall time period.**	**Summary results in percentage with guidance notes**	**User can select additional support if results are more problematical**	**Simple links page with URL connection or ability to add new close friends etc**
**Effects / animation / sound**	**SFX; Thoughtful hmmmm sounds**<br>**Animation; Slight movement to mouth.**				

8. OPTIONS

Home page | **Colour ways** | **Landscape option**

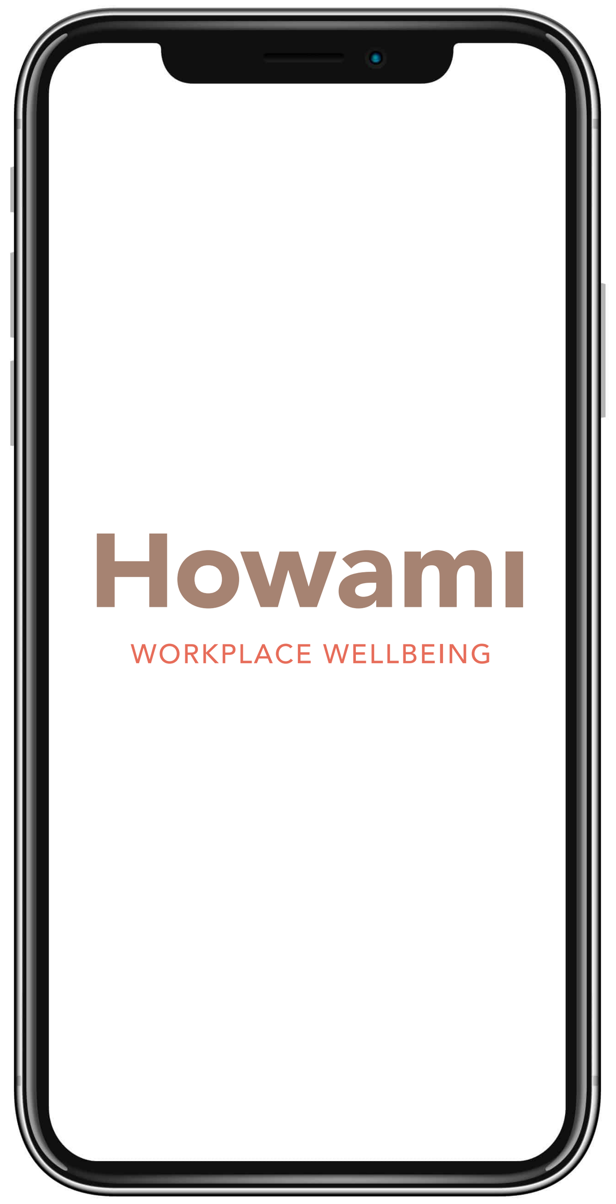

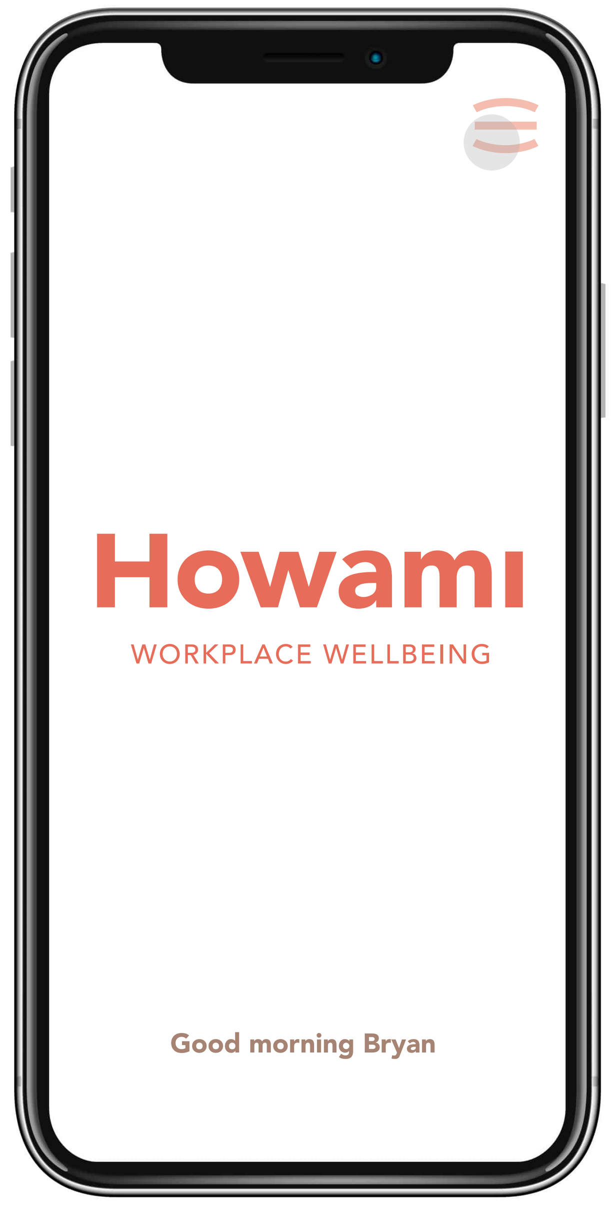

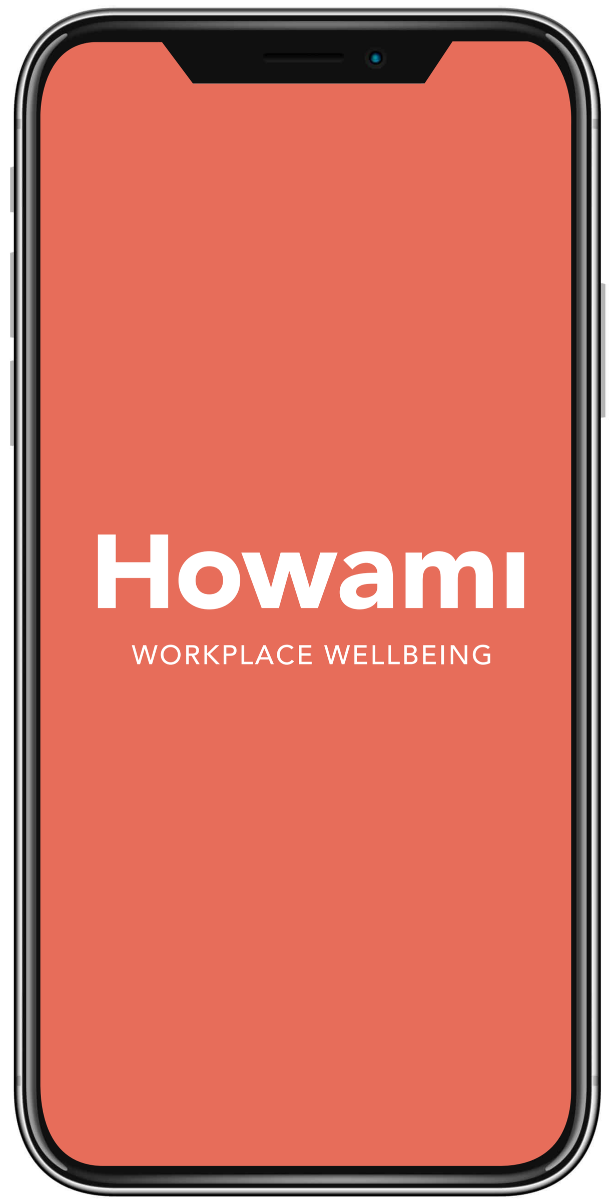

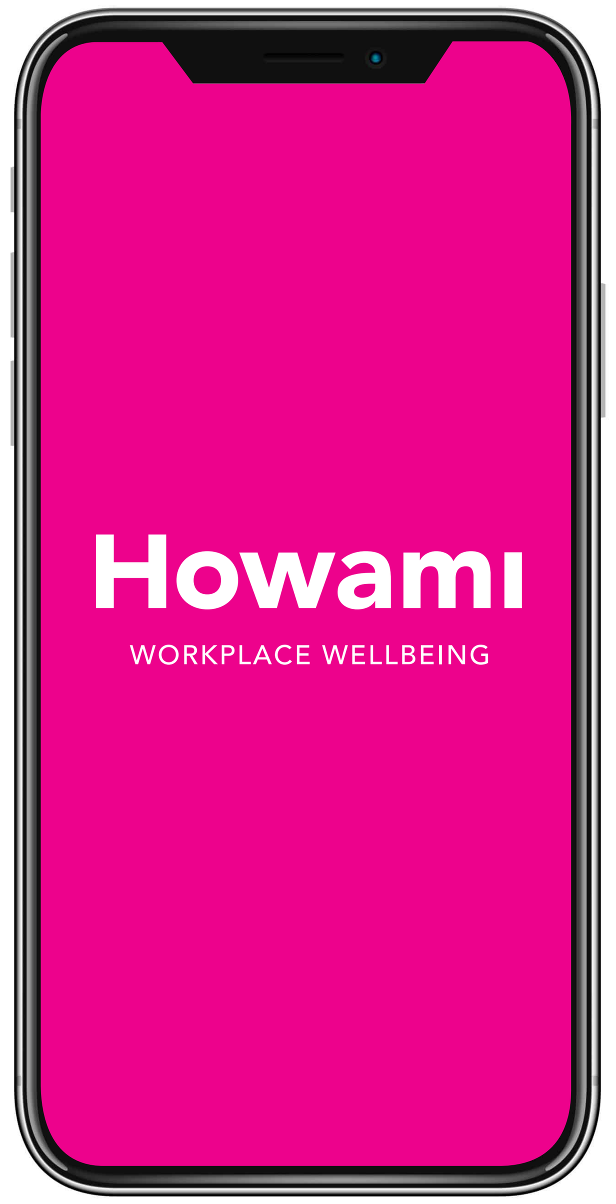

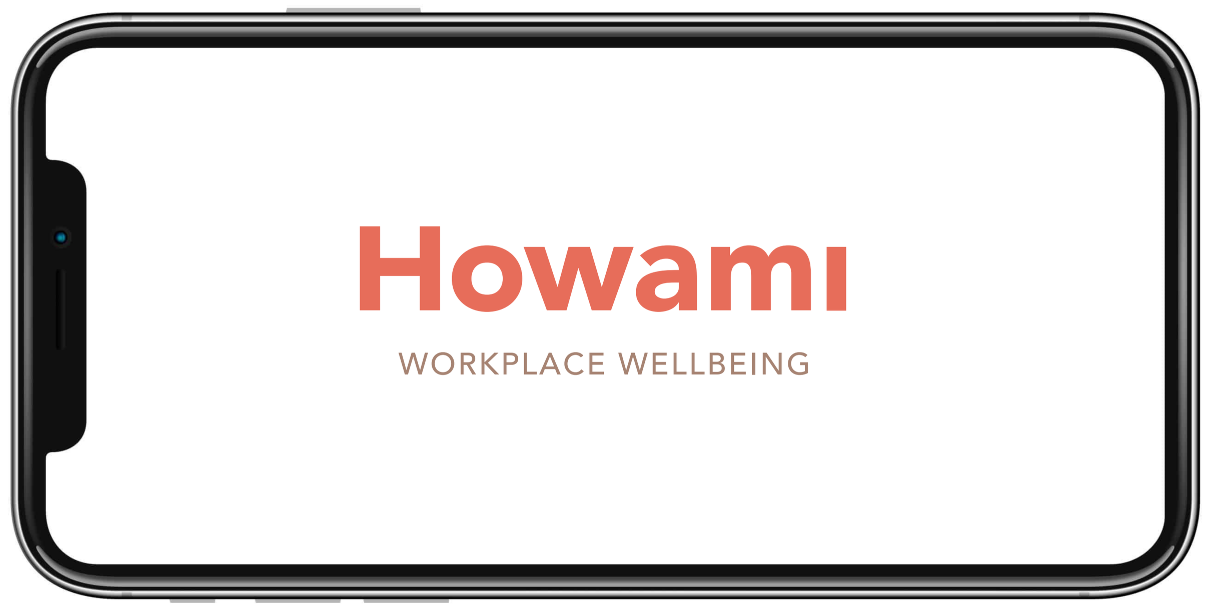

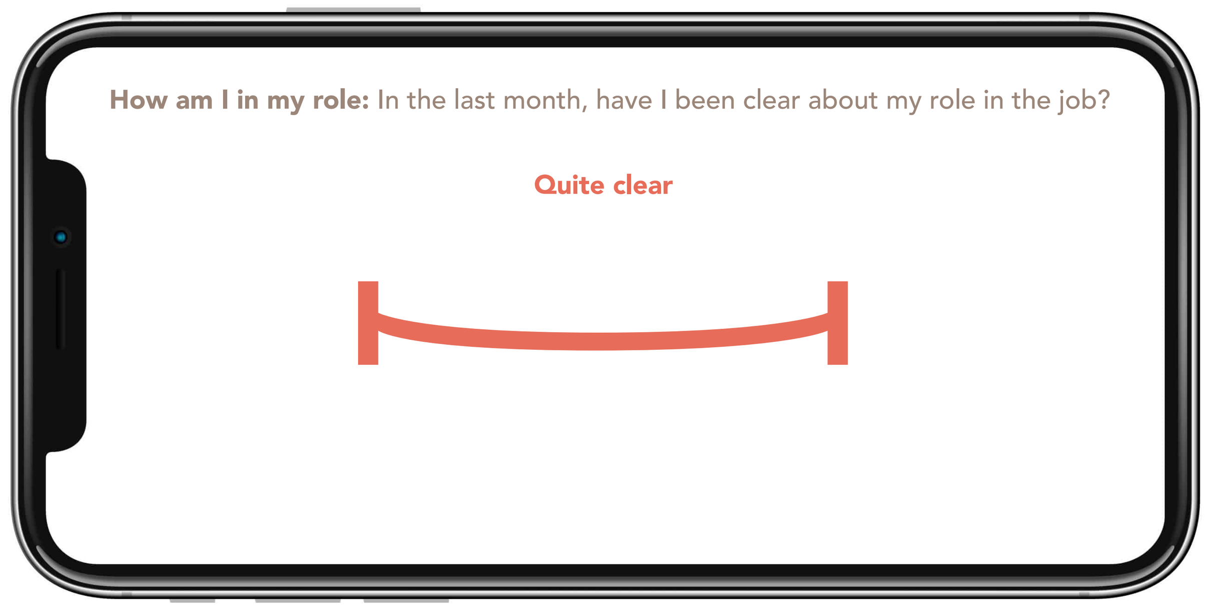

Supplement: Supplementary file 4 — Supporting Information 4 [file HTL2-12-e70009-s003.pdf]
